# Stress-Driven Discovery of Novel Cryptic Antibiotics from a Marine Fungus *Penicillium* sp. BB1122

**DOI:** 10.3389/fmicb.2017.01450

**Published:** 2017-08-03

**Authors:** Bibi N. Auckloo, Chengqian Pan, Najeeb Akhter, Bin Wu, Xiaodan Wu, Shan He

**Affiliations:** ^1^Ocean College, Zhejiang University Hangzhou, China; ^2^Center of Analysis and Measurement, Zhejiang University Hangzhou, China; ^3^Laboratory of Marine Natural Products, School of Marine Sciences, Ningbo University Ningbo, China

**Keywords:** marine fungus, metal-stress strategy, spectroscopic techniques, polyketide, antibiotics

## Abstract

Standard laboratory cultures have long been known to hinder activation of specific gene clusters which in turn hamper production of secondary metabolites with unique properties due to lack of innovation or the inability to trigger cryptic gene clusters’ expression. Due to challenges related to the avoidance of the isolation of replicated metabolites, resistance-developing pathogens are to be addressed by the scientific community worldwide in order to progress with novel and potent compounds which could further be developed in the future for pharmaceutical usage. This study reports the isolation of novel cryptic antibiotics from a marine fungus *Penicillium* sp. BB1122 collected from Zhoushan coast by applying the “metal-stress” strategy, here referring to the heavy metal cobalt (6 mM). High-performance liquid chromatography-guided isolation of four novel and four known compounds belonging to the polyketide class has been carried out where their relative as well as absolute configurations have been determined using spectroscopic analysis techniques as well as by the comparison of theoretically calculated ECD spectrum and the experimental ECD spectrum, respectively. The structures of novel compounds **7** and **8** represent the first example of 2,5-dioxabicyclo[2.2.1]heptane pyrone backbone bearing a migrated polyene chain. The novel compounds **7, 8**, and **5** exhibited impressive antibiotic properties against methicillin resistant *Staphylococcus aureus* (MRSA) with MIC value of around 0.5 and 1 μg/mL, respectively. Moreover, the new compounds **1, 7**, and **8** displayed potent antibiotic activities with MIC values of around 4 μg/mL against the pathogenic *Pseudomonas aeruginosa*. Moreover, the MBC of the different potent compounds ranged from 1 to 128 μg/mL against MRSA, *P. aeruginosa*, and *Klebsiella pneumoniae*. In addition, the cytotoxic activities were also evaluated where new antibiotics **7** and **8** were not obviously harmful toward normal liver cell lines LO2, showing IC_50_ values above 100 μg/mL. As a consequence, the results from this study unveiled that cobalt stress is an effective strategy to discover novel antibiotics from microorganisms.

## Introduction

The marine biosphere is known to be a treasure of unique, priceless, and potent biologically active natural products which originate from both marine flora and fauna (Ammerman et al., 1984; Kang et al., 2015). Marine organisms usually strive under specific conditions like temperature, pressure, dissolved oxygen, and nutrient availability, thus leading to the generation of a structural miscellany and biochemical distinctiveness of secondary metabolites compared to terrestrial ones (Aneiros and Garateix, 2004; Bhatnagar and Kim, 2010; Barboza et al., 2012; Martin et al., 2013; Tsoukalas et al., 2014; Rangnekar and Khan, 2015). As such, reports by Blunt et al. (2017) unveiled a total of 14,637 from 2001 to 2015 in the isolation of novel natural products from marine organisms. Marine microorganisms have proved themselves in producing compounds with tremendous activities like antibacterial properties (Lincke et al., 2010), antitumor capacities (Kwon et al., 2006), or anticancer abilities (Luesch et al., 2001; Gomes et al., 2015). More specifically, marine fungi-derived compounds have demonstrated their abilities to exhibit antibacterial effects in previous studies such as Terretonin G isolated from *Aspergillus* sp. OPMF00272 in Japan (Fukuda et al., 2014) as well as several tryptoquivalines and meroditerpenes also isolated from various marine fungi like *Neosartorya paulistensis, Neosartorya laciniosa*, and *Neosartorya tsunodae* (Gomes et al., 2014). More useful information can be reviewed in literature (Auckloo and Wu, 2016; Imhoff, 2016). Moreover, the use of diverse biotechnological techniques has revolutionized the scientific world by significantly contributing to the production of various products for pharmaceutical purposes such as lifesaving drugs. Marine biotechnology had great effects on chemical products or enzymes as well as pharmaceutical drugs such as ziconotide (painkiller) isolated from cone snail, Ara-C (anticancer) isolated from sponge or the green fluorescent protein isolated from the jelly fish *Aequorea victoria* to name a few (Thakur and Thakur, 2006; Kumar Gupta et al., 2015). Moreover, marine fungi have been revealed as possessing tremendous antibiotic properties where their discovery till their production had been discussed (Silber et al., 2016). It is true that various compounds are actually in different clinical phases, however, the industrial scale production of potent natural products should not be neglected where a biotechnological approach would also be among the utmost priorities. Due to the lack of valid resources in terms of funding or manpower, strategies and/or technologies, an incessant decline in isolation of effective antibacterial compounds were seen during the past years. Moreover, owing to the evolutionary concept, pathogens had the ability to rapidly mutate resulting in the amendment of their genetic materials leading to resistance capacities. Therefore, urgent innovative techniques are a must to isolate and elucidate potent novel candidates with antibiotic properties. According to Shi et al. (2015), generation of natural products can be intensified by tolerant microorganisms dwelling in heavy metal stressed environment by chelation capacities. Based on a previous study, the hypothesis of “metabolism switching” can be put forward in relation to secondary metabolites production from tolerant strains under stressed conditions (Iwai and Omura, 1982). As such, novel and potent metabolites production may be influenced by specific chelating capabilities, distinct enzymatic reactions as well as unlocking of cryptic biosynthetic gene clusters in microbes.

This paper introduces the “metal-stress” concept on a marine fungus isolated from marine sediment along Zhoushan Coast, China. In this experiment, the “metal-stress” strategy referred to the concept of applying specific concentrations of particular heavy metals which would act as an elicitor to the culture of *Penicillium* sp. BB1122 strain, with the high probability of unlocking cryptic gene clusters, thus inducing the production of novel secondary metabolites. The isolation and structure elucidation of several isolated novel polyketides compounds are discussed followed by their antibiotic activity screening against methicillin resistant *Staphylococcus aureus* (MRSA), *Pseudomonas aeruginosa*, and *Klebsiella pneumoniae*.

## Materials and Methods

### General Experimental Procedures

The high-performance liquid chromatography (HPLC) system used was composed of a Waters 717 plus Autosampler, a Waters 600 Controller, a Waters 996 Photodiode Array Detector, and a Waters Millog workstation (Waters, Shinagawa, Tokyo, Japan). Optical rotations were measured in methanol on a PerkinElmer-341 polarimeter. The IR spectra were run on a NicoletAvatar-360FT-IR spectrometer. ^1^H NMR (500 MHz) and ^13^C NMR (125 MHz) spectra were measured at 25°C on a Bruker AVANCE DMX 500 NMR spectrometer with TMS as internal standard. CD spectra were measured on a JASCO J-715 (JASCO) spectropolarimeter. UV spectra were also recorded in methanol on a Shimadzu UV2550. ESIMS were recorded on an Agilent 6460 Triple Quad LCMS. Preparative HPLC was performed on a ChuangXinTongHeng system equipped with a Venusil MP-C18 column (10 mm × 250 mm, Agela Technologies, Tianjin, China). The organic solvents used in chromatographic separation were of analytical grade purchased from Sayfo Technology (Tianjin China) and chromatographic grade for HPLC analysis purchased from Tedia, United States. Deionized water was prepared by reverse osmosis Milli-Q water (18 MW; Millipore, Bedford, MA, United States) and used for all solutions and dilutions. Agar powder for plate culture and cobalt chloride was purchased from Sinopharm Chemical Reagent Co., Ltd. (Shanghai, China).

### Isolation, Normal and Metal-Stress Cultivation of the Fungal Strain

#### Isolation of the Fungal Strain

The fungus was isolated from marine sediment collected along Zhoushan coast, China in January 2016. The latter was grown on PDA agar, consisting of 100 g potato lixivium, 10 g dextrose, 35 g sea salt, and 15 g agar powder per liter. During this study, the fungus strain was viewed under the microscope where the conidiophore was seen as being branched. Conidia were glued with phialides which in turn were attached at the end of conidiophores (Houbraken and Samson, 2011; Samson et al., 2011). According to the morphology of the strain as well as on the basis of the ITS 18S fragment, *Penicillium* sp. BB1122 was ascribed to the fungus (see Supplementary Table S2).

#### Normal and Metal-Stress Cultivation

The normal cultivation of *Penicillium* sp. BB1122 was carried out in 500 mL flasks containing 200 mL liquid PDB for 14 days at 28°C under static conditions. Then the metal-stress method was applied using six different metals namely cobalt, manganese, chromium, nickel, zinc, and cadmium with original concentrations 100, 200, 400, and 800 μM each, respectively. The mycelium was removed and culture broth was extracted with an equal volume of ethyl acetate. Due to the unchanged HPLC profile of the normal and stressed cultures, the concentrations of the different metals were increased where the final optimal stress conditions was determined as 6 mM cobalt ion concentration for stress culture based on the HPLC profile of the extract and the peak areas of the stress-induced products. Moreover, both the normal and cobalt-stressed cultures were tested for their antibacterial capacities which enhanced our decision to enlarge 6 mM cobalt culture due to the stressed culture’s effective inhibitory abilities.

#### Fermentation in Large Scale

*Penicillium* sp. BB1122 was inoculated onto agar plates containing PDA medium. After incubation for 14 days at 28°C, the pre-culture was used for inoculation in two 500 mL flasks containing 200 mL liquid medium as parent bottles. The parent bottles were incubated for 14 days at 28°C under static conditions. This broth was used to amplify the culture scale. A total of 30 L of broth containing 6 mM cobalt ions was used for large scale fermentation to give an organic extract of 3.58 g.

### Extraction, HPLC-Guided Isolation, and Identification of Stress Metabolites

The 30 L of fermentation broth was extracted with ethyl acetate. After evaporation of the solvent the crude extract was dissolved in methanol. Analytical reversed phase HPLC-UV experiments were performed using a C_18_ column (sepax Amethyst C_18_-H, 100 mm 3.00 mm) applying a water/methanol gradient from 20% methanol to 100% methanol in 30 min, maintaining 100% methanol for 10 min, flow rate 0.8 mL/min on a LC20A system (Shimadzu, Kyoto, Japan) equipped with a Prominence CBM-20A/20 Alite controller, SPD-20A UV detector, and Prominence CTO-20A column oven. The stress-induced compounds were isolated by preparative HPLC, which was carried out using a HPLC-UV system (P3000 type high pressure infusion pump, UV3000 type ultraviolet/visible light detector, Rheodyne 7725 I manual sampling valve, sepax C_18_, 150 mm 80 mm, column). Based on the results of the analytical HPLC analysis, gradient phase of 40–100% methanol was applied for 2 h for isolation of compounds **1–8** at a flow rate of 10 mL/min.

*Neocitreoviridin (****1****)*: yellowish syrup; [α]^24^_D_ + 22.3 (*c* 0.25, CHCl_3_); UV (MeOH) λ_max_ (log *𝜖*) 250 (4.02), 301 (3.92) nm; IR ν_max_ 3439, 3089, 2973, 1731, 1557, 1401, 1254, 1137, 1089, 972, 867, 811, 624, 602 cm^-1^; ^1^H NMR and ^13^C NMR, see **Table 1**; HR-TOF-MS *m/z* 425.1919 [M+Na]^+^ (calcd. for C_23_H_29_NaO_6_, 425.1935).

**Table 1:** ^1^H NMR data (500 MHz, δ in ppm, *J* in Hz), ^13^C NMR data (125 MHz, δ in ppm) for compounds **1** and **2** in CDCl_3_.

Position	1 (CDCl_3_)		2 (CDCl_3_)	
	δ_C_	δ_H_ (*J* in Hz)	δ_C_	δ_H_ (*J* in Hz)
1	12.46, CH_3_	1.19d (6.36)	12.26, CH_3_	1.18d (6.10)
2	77.51, CH	3.85^∗^	77.50, CH	3.84^∗^
3	81.26, C		80.79, C	
4	84.74, CH	3.99s	85.60, CH	3.98s
5	86.50, C		84.10, C	
6	148.17, CH	5.72s	141.41, CH	5.50s
7	136.29, C		136.11, C	
8	138.49, CH	6.37^∗^	140.84, CH	6.36^∗^
9	127.04, CH	6.35^∗^	127.75, CH	6.34^∗^
10	139.35, CH	6.59dd (14.53, 11.04)	138.71, CH	6.51^∗^
11	130.26, CH	6.32^∗^	130.96, CH	6.47dd (16.51, 12.11)
12	136.47, CH	7.20^∗^	134.07, CH	7.18dd (14.54, 11.57)
13	119.15, CH	6.34^∗^	118.43, CH	6.39^∗^
14	154.93, C		154.60, C	
15	108.51, C		107.84, C	
16	171.39, C		170.85, C	
17	88.82, CH	5.57s	88.40, CH	5.50s
18	164.97, C		164.19, C	
19	17.49, CH_3_	1.20s	17.37, CH_3_	1.22s
20	22.66, CH_3_	1.39s	21.27, CH_3_	1.38s
21	14.15, CH_3_	1.94s	13.30, CH_3_	1.92s
22	9.11, CH_3_	1.98s	9.18, CH_3_	1.96s
23	56.57, CH_3_	3.85s	56.16, CH_3_	3.84s

*^∗^Peaks are overlapped.*

*10Z-isocitreoviridinol (****5****)*: yellowish syrup; [α]^24^_D_ - 17.2 (*c* 0.18, CHCl_3_); UV (MeOH) λ_max_ (log *𝜖*) 268 (3.98), 360 (3.95) nm; IR ν_max_ 3399, 2969, 1708, 1655, 1629, 1560, 1452, 1400, 1252, 1144, 1089, 1020, 938, 811 cm^-1^; ^1^H NMR and ^13^C NMR, see **Table 2**; HR-TOF-MS *m/z* 419.2049 [M+H]^+^ (calcd. for C_23_H_31_O_7_, 419.2064).

**Table 2:** ^1^H NMR data (500 MHz, δ in ppm, *J* in Hz), ^13^C NMR data (125 MHz, δ in ppm) for compounds **5** and **6** in MeOD.

Position	5 (MeOD)		6 (MeOD)	
	δ_C_	δ_H_ (*J* in Hz)	δ_C_	δ_H_ (*J* in Hz)
1	13.49, CH_3_	1.16d (6.43)	13.49, CH_3_	1.17^∗^
2	80.97, CH	4.04^∗^	80.24, CH	4.08^∗^
3	84.76, C		86.00, C	
4	76.51, CH	3.98s	75.85, CH	3.99s
5	84.50, C		84.96, C	
6	80.97, CH	3.60s	82.25, CH	3.75s
7	79.82, C		79.26, C	
8	148.53, CH	6.10^∗^	147.54, CH	6.21^∗^
9	128.71, CH	6.41^∗^	127.36, CH	6.39^∗^
10	138.87, CH	6.57^∗^	139.53, CH	6.59^∗^
11	132.49, CH	6.49^∗^	132.00, CH	6.46^∗^
12	137.05, CH	7.14dd (14.98, 10.99)	137.12, CH	7.12dd (15.39, 11.41)
13	120.36, CH	6.55^∗^	120.01, CH	6.54^∗^
14	155.90, C		156.00, C	
15	109.82, C		109.61, C	
16	173.12, C		173.10, C	
17	89.14, CH	5.62s	89.06, CH	5.60s
18	166.37, C		166.37, C	
19	17.52, CH_3_	1.27s	16.70, CH_3_	1.30s
20	18.65, CH_3_	1.29s	17.29, CH_3_	1.30s
21	26.72, CH_3_	1.26s	27.88, CH_3_	1.35s
22	8.87, CH_3_	2.00s	8.89, CH_3_	1.99s
23	57.28, CH_3_	3.90s	57.27, CH_3_	3.89s

*^∗^Peaks are overlapped.*

*Penicillstressol (****7****)*: yellowish syrup; [α]^24^_D_ - 35.9 (*c* 0.20, CHCl_3_); UV (MeOH) λ_max_ (log *𝜖*) 274 (3.90) nm; IR ν_max_ 3150, 1711, 1608, 1560, 1515, 1402, 1256, 1169 cm^-1^; ^1^H NMR and ^13^C NMR, see **Table 3**; HR-TOF-MS *m/z* 419.2045 [M+H]^+^ (calcd. for C_23_H_31_O_7_, 419.2064).

**Table 3:** ^1^H NMR data (500 MHz, δ in ppm, *J* in Hz), ^13^C NMR data (125 MHz, δ in ppm) for compounds **7** and **8** in MeOD.

Position	7 (MeOD)		8 (MeOD)	
	δ_C_	δ_H_ (*J* in Hz)	δ_C_	δ_H_ (*J* in Hz)
1	13.56, CH_3_	1.13^∗^	13.03, CH_3_	1.21^∗^
2	16.69, CH	1.25^∗^	13.43, CH	1.27^∗^
3	85.19, C		80.27, C	
4	78.70, CH	3.70s	78.51, CH	3.69s
5	87.96, C		85.90, C	
6	91.10, CH	4.18s	89.28, CH	4.17s
7	137.70, C		136.55, C	
8	128.70, CH	6.18d (11.39)	127.55, CH	6.18d (10.90)
9	130.99, CH	6.54^∗^	130.86, CH	6.56^∗^
10	133.62, CH	6.28^∗^	131.39, CH	6.30^∗^
11	134.34, CH	6.47^∗^	133.49, CH	6.46dd (14.88, 10.81)
12	131.54, CH	5.91dd (15.10, 6.83)	128.72, CH	5.90^∗^
13	70.30, CH	5.24d (6.85)	70.13, CH	5.24d (6.96)
14	160.41, C		160.25, C	
15	109.64, C		109.50, C	
16	173.37, C		173.22, C	
17	89.42, CH	5.62s	88.01, CH	5.62s
18	166.85, C		166.72, C	
19	13.16, CH_3_	1.27s	12.77, CH_3_	1.20s
20	13.81, CH_3_	1.16s	15.42, CH_3_	1.13s
21	15.56, CH_3_	1.78s	13.69, CH_3_	1.77s
22	8.78, CH_3_	2.00s	8.67, CH_3_	2.00s
23	57.47, CH_3_	3.90s	57.35, CH_3_	3.82s

*^∗^Peaks are overlapped.*

*Isopenicillstressol (****8****)*: yellowish syrup; [α]^24^_D_ - 42.3 (*c* 0.10, CHCl_3_); UV (MeOH) λ_max_ (log *𝜖*) 274 (3.93) nm; IR ν_max_ 3415, 1711, 1656, 1631, 1561, 1453, 1400, 1286, 1253, 1213, 1090, 1063, 1020, 939, 812 cm^-1^; ^1^H NMR and ^13^C NMR, see **Table 3**; HR-TOF-MS *m/z* 419.2048 [M+H]^+^ (calcd. for C_23_H_31_O_7_, 419.2064) (see Supplementary Material).

### Computation Section

The geometry was optimized starting from initial conformations, with DFT calculations at the B3LYP/6-31+G(d) level using the Gaussian 09 program. Frequency analysis was done at the same level of theory to verify that these optimized structures are real minima on the potential energy surface. Time-dependent DFT calculations were performed on the lowest-energy conformations for each configuration using 30 excited states and under the methanol solution. ECD spectra were generated using the program SpecDis by applying a Gaussian band shape with 0.2 eV width, from dipole-length rotational strengths (Frisch et al., 2009; Xie et al., 2011, 2012; Bruhn et al., 2015).

### Antibiotic Activity Screening of Stress Metabolites

The conventional broth dilution assay was used to evaluate the antibacterial activities. Three clinical pathogens namely MRSA, *P. aeruginosa* [CMCC(B)10104], and *K. pneumoniae* [CMCC(B)46117] were cultured and left overnight to grow. Each pathogenic culture was then diluted in 0.9% saline to an inoculum density of 5 × 10^5^ cfu by comparison with a McFarland standard. Tetracycline was dissolved in Mueller–Hinton broth with a starting concentration of 512 μg/mL and was used as positive control whereas methanol was used as negative control. A total of 125 μL of MHB was distributed into the 96-well plates. A total of 125 μL of the different samples were dispensed into well 1 and serially diluted across the plate. Ultimately, addition of 125 μL of the bacterial inoculum was performed and the plates were incubated at 37°C for 18 h. The bacteriostatic abilities of the compounds were noted as MICs which were done in triplicate. Five milligrams per milliliter of methanolic solution of 3-[4,5-dimethylthiazol-2-yl]-2,5-diphenyltetrazolium bromide (MTT; Lancaster) was used to detect bacterial growth by a change in color from yellow to blue (Appendino et al., 2008).

Ten microliters of broth with no visible growth were taken from the wells and sub-cultured on nutrient agar plates. The negative control consisted of nutrient agar and the inoculum which was then compared with the number of colonies on the agar. The bactericidal abilities of the compounds were noted as MBCs which was equal to the lowest concentration of the samples which killed 99.9% of the original inoculum (Clinical and Laboratory Standards Institute, 2014).

### Cytotoxicity Assay

Human liver cell line LO2 was obtained from the Institute of Biochemistry and Cell Biology (Chinese Academy of Sciences). LO2 cells were maintained in RPMI 1640 containing 10% fetal bovine serum (FBS), 100 units/mL penicillin and 100 units/mL streptomycin. All the cells were grown in a humidified cell incubator with an atmosphere of 5% CO_2_ at 37°C and subcultured with 0.25% trypsin + 0.02% EDTA. MTT, dimethyl sulfoxide (DMSO), ribonuclease (RNase), propidium iodide (PI), Annexin V-FITC/PI kit were obtained from Sigma. RPMI 1640, DMEM, FBS were purchased from Gibco. Hoechst, trypsin, and the pan caspase inhibitor z-VAD-FMK were obtained from Beyotime Institute of Biotechnology. Compounds were dissolved in DMSO and diluted to the proper concentrations before use, with the concentration of DMSO kept below 0.1% in all assays. Cell viability was measured by the MTT method as previously described, with some modifications (Jiang et al., 2013). Briefly, cells were seeded in 96-well microtiter plates at a density of 5 × 10^3^ cells/well for 24 h. After drug treatment for the indicated times, cells were incubated with MTT (0.5 mg/mL) for 4 h. The formazan precipitate was dissolved in 150 μL DMSO, and the absorbance was detected at 490 nm with a Sunrise microplate reader (Tecan Group Ltd.). DMSO (0.1%) was used as negative control for all assays. Each test was performed in triplicate.

## Results and Discussion

During this study, cobalt was chosen as the elicitor to stress the fungus which boosted up its antibacterial exhibition in comparison to the normal growing strain. To prove this hypothesis, the MIC of the cobalt-stressed extract was tested against the three pathogens namely MRSA, *P. aeruginosa*, and *K. pneumoniae* where it was revealed to be much lower (0.5 μg/mL) compared to the normal culture extract with much higher MIC value which was considered negligible.

**FIGURE 1:**
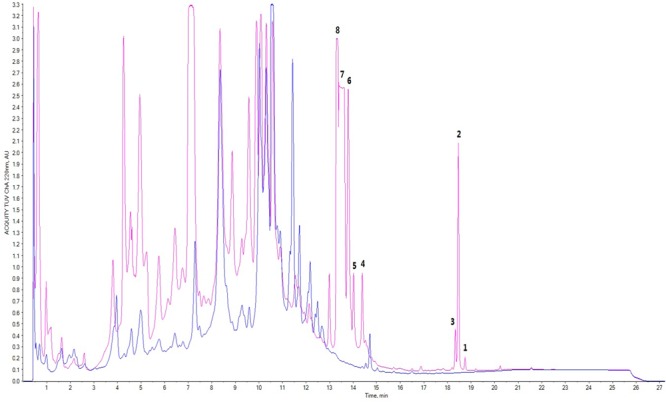
Comparison of HPLC profile of normal culture (blue) and cobalt-stressed culture (pink); peaks 1–8 represented stress metabolites **1–8** where **1, 5, 7**, and **8** represented novel compounds whereas **4, 6, 3**, and **2** were known compounds.

**FIGURE 2:**
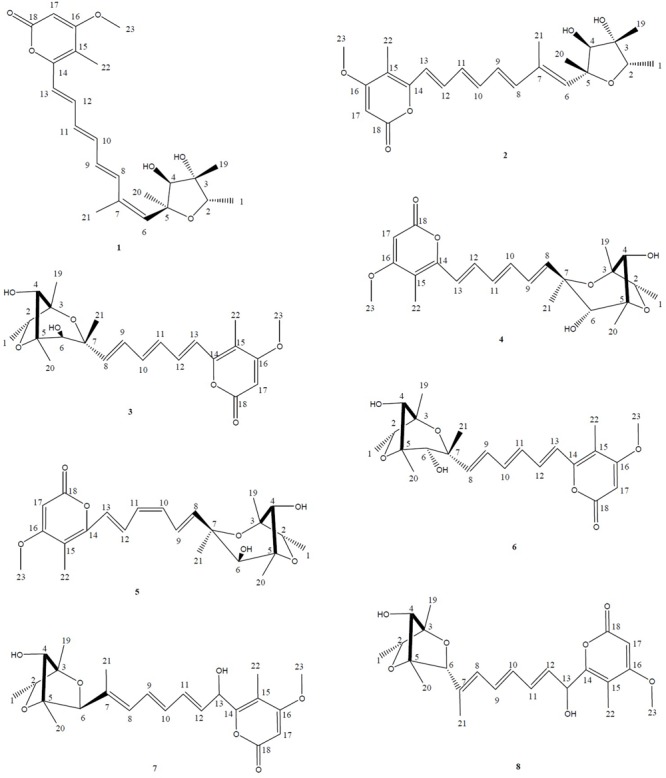
Structures of the isolated stress metabolites **1–8**. HPLC-guided isolation of four novel compounds corresponding to a citreoviridin analog **(1)**, a new polyene pyrone polyketide with a 2,6-dioxabicyclo[3,2,1]octane ring **(5)** and two new polyene pyrone polyketides with 2,5-dioxabicyclo[2.2.1]heptane ring (**7** and **8**) together with four known compounds epiisocitreoviridinol **(4)**, citreoviridinol **(6)**, epicitreoviridinol **(3)**, and citreoviridin **(2)** were isolated from cobalt-stressed *Penicillium* sp. BB1122.

Moreover, based on the difference of the HPLC profile between the normal culture (blue) and the cobalt-stressed culture (pink), eight metabolites which as shown in **Figure 1** have been successfully isolated, among which four are novel and four are known compounds. In this study, our focus pointed out on the semi-polar compounds which were easily identified as new peaks popping out compared to their absence in the normal culture. As such, enlargement of cobalt-stressed culture was performed following the HPLC-guided isolation which allowed the isolation of the stress metabolites by application of the preparative HPLC (C_18_) followed by the elucidation and confirmation of a new citreoviridin analog (**1**), a new polyene pyrone polyketide with a 2,6-dioxabicyclo[3,2,1]octane ring (**5**) and two new polyene pyrone polyketides with 2,5-dioxabicyclo[2.2.1]heptane ring (**7** and **8**) together with four known compounds with the help of spectroscopic analysis techniques like 1D, 2D NMR and HRMS (**Figure 2**).

### Novel Compounds Elucidation

#### Compound 1

**FIGURE 3:**
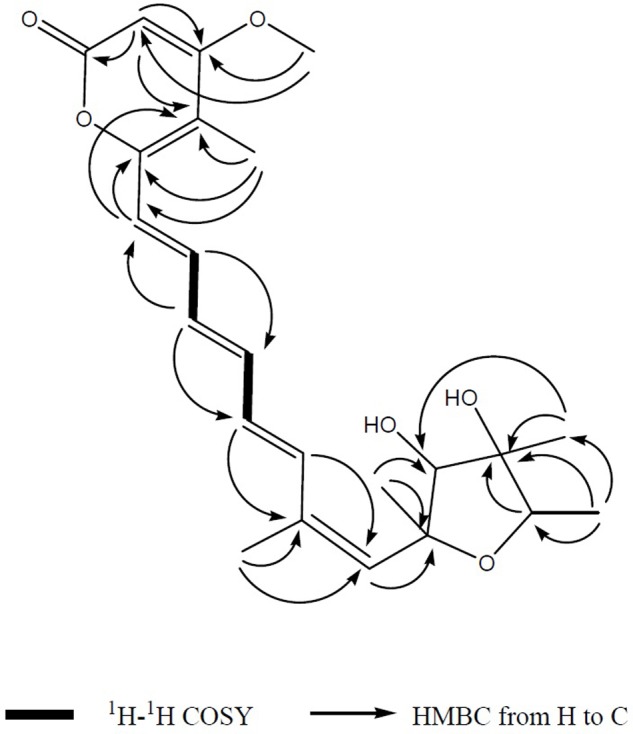
The key ^1^H-^1^H COSY and HMBC correlations of compound **1**. The connection of the major three domains namely an α-pyrone ring and a tetrahydrofuran ring connected by an olefinic chain was mainly determined after interpretation of 1D and 2D NMR spectrum.

**FIGURE 4:**
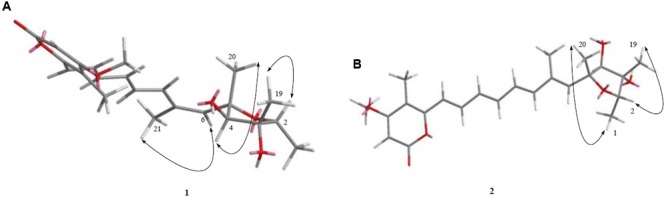
**(A,B)**. The key and NOESY correlations of compounds **1** and **2**. **(A)** NOESY cross peaks of H-6/Me-21, H-4/Me-20, and H-2/Me-19 were seen after analysis of 2D NMR spectrum. **(B)** NOESY cross peaks of Me-1/Me-20, H-2/Me-19 was observed after analysis of 2D NMR spectrum.

Compound **1** was isolated as a yellowish syrup. The molecular formula was determined to be C_23_H_30_O_6_ by analysis of the HR-TOF-MS ion peaks at *m/z* 425.1919 [M+Na]^+^ (calcd. 425.1935), *m/z* 403.2102 [M+H]^+^ (calcd. 403.2115), and *m/z* 827.3964 [2M+Na]^+^ (calcd. 827.3977). The ^1^H and ^13^C NMR of **1** (**Table 1**) showed similar chemical shifts and same multiplicities for 23 carbon atoms of a tetrahydrofuran ring, an α-pyrone ring and an octa-1,3,5,7-tetraene with a minor difference indicating that compound **1** supposedly possess the basic structure of α-pyrone type polyketide (da Rocha et al., 2015). Naturally occurring α-pyrone type polyketides can be categorized into two main classes: verrucosidinol analogs and citreoviridin analogs. Both classes of molecules contain two distinct domains: α-pyrone ring and a tetrahydrofuran ring connected by an olefinic chain (Pan et al., 2016). Both polyketides analogs exist in nature with the verrucosidinol analogs found in the majority of structures. The main differences lie in the olefinic chain where there are three methyl groups on the olefinic chain of verrucosidinol analogs whereas only one methyl group is found on the olefinic chain of citreoviridin analogs. The characteristic olefinic proton signals at δ_H_ 5.72 (s, H-6), 6.37 (H-8), 6.35 (H-9), 6.59 (dd, *J* = 14.53, 11.04 Hz, H-10), 6.32 (H-11), 7.20 (H-12), 6.34 (H-13), and a methyl protons signal at δ_H_ 1.94 (s, H-21) in the ^1^H NMR spectrum excluded the possibility of the existence of verrucosidinol analogs. This influence was further confirmed by the lack of the substructure of oxirane ring in **1**. The diagnostic sequence of H8 = H9 = H10 = H11 = H12 = H13 deducted from the cross peak of H-9/H-10, H-10/H-11, H-11/H-12, H-12/H-13 in the ^1^H-^1^H COSY of **1** demonstrated the presence of citreoviridin backbone. To clarify the connection of three domains and the positions of two hydroxyls, five methyls, one carbonyl, and a methoxy group in the supposed citreoviridin backbone, detailed 2D NMR experiments were carried out. The olefinic protons signals at δ_H_ 5.57 (s, H-17) showed long range correlations with lactone carbonyl carbon signal at δ_C_ 164.97 (C, C-18) and δ_C_ 108.51 (C, C-15) and δ_C_ 171.39 (C, C-16), among which the olefinic C-16 displayed HMBC cross peak of OCH_3_/C-16, positioning the carbonyl at C-18 and OCH_3_ at C-16 of the α-pyrone ring. The Me-22 was located at C-15 owing to the long range correlation between δ_H_ 1.98 (s, Me-22) and δ_C_ 154.93 (C, C-14), 119.15 (CH, C-13) in the HMBC spectrum of **1**. The HMBC cross peak of Me-21/C-7 and Me-21/C-6 and NOESY cross peak of H-6/Me-21 were observed, exhibiting that Me-21 was assigned to C-7 on the olefinic ring in molecule **1**. Two oxygenated tertiary carbons at δ_C_ 77.51 (CH, C-2), 84.74 (CH, C-4) and two oxygenated quaternary carbons at δ_C_ 81.26 (C, C-3), 86.50 (C, C-5) were observed in the ^13^C NMR spectrum of **1**, revealing the presence of a highly oxygenated tetrahydrofuran ring. Three methyl groups on the tetrahydrofuran ring positioned at C-2, C-3, and C-5 were deducted from the observation of long range correlations from proton signal at δ_H_ 1.19 (d, *J* = 6.36 Hz, Me-1) to carbon resonance at δ_C_ 77.51 (CH, C-2), 81.26 (C, C-3), and correlations from proton resonance at δ_H_ 1.20 (s, Me-19) to carbon signals at δ_C_ 77.51 (CH, C-2), 84.74 (CH, C-4), and correlations from the proton signals at δ_H_ 1.39 (s, Me-20) to carbon resonance at δ_C_ 84.74 (CH, C-4), 86.50 (C, C-5). Two hydroxyl groups were inferred from the oxygenated carbon resonance feature of C-3 and C-4 in the ^13^C NMR spectrum of **1** with assistance to the molecular formula C_23_H_30_O_6_. The α-pyrone ring and the olefinic chain were bridged by C13–C14 bond deducted from the HMBC analysis of the cross peaks from hydrogen signal at δ_H_ 6.34 (H-13) to carbon resonances at δ_C_ 154.93 (C, C-14), 108.50 (C, C-15) and hydrogen signal at δ_H_ 1.98 (s, Me-22) to carbon signal at δ_C_ 119.15 (CH, C-13). The long range correlation ship from hydrogen signal at δ_H_ 5.72 (s, H-6) to carbon signal at δ_C_ 86.50 (C, C-5) proved the linkage of C5–C6 between the tetrahydrofuran ring and the olefinic chain, which was also confirmed by NOESY cross peak C-6/C-4 in compound **1**. From the above analysis, the planar structure was elucidated as drawn in **Figure 3** which appeared the same as citreoviridin. Detailed analysis of NOESY experiment (**Figure 4**) proved that the geometry of **1** of C6=C7 double bond was different from the *E* configuration of C6=C7 in citreoviridin. The diagnostic NOESY correlation from proton signal at δ_H_ 5.72 (s, H-6) to proton signal at δ_H_ 1.94 (s, Me-21) demonstrated *Z* configuration of C6=C7 which is the first example in nature. The relative configuration at C-5 was proved to be different from that of the known compound citreoviridin. In the NOESY spectrum of **1**, the proton resonance at δ_H_ 1.39 (s, Me-20) showed cross peak with proton signal at δ_H_ 3.99 (s, H-4) indicating that the olefinic chain at C-5 and the hydroxyl at C-4 were on the same side as β-oriented as opposed to the α-oriented of the olefinic chain in the known compound citreoviridin. The hydroxyl at C-3 and the methyl at C-2 were on the same side due to the observation of the NOESY cross peak of Me-19/H-2, and at the same time Me-19 showed no correlation with H-4 indicating an α-oriented Me-1 and β-oriented Me-19.

**FIGURE 5:**
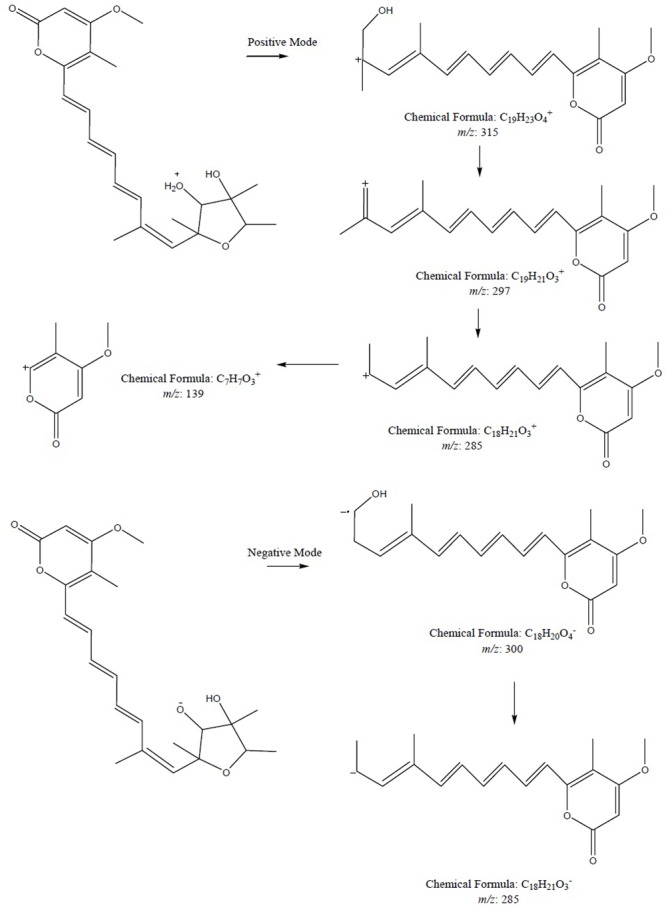
The plausible key MS^2^ fragmentation pathway of compound **1** was confirmed by ESI-MS/MS analysis in positive and negative modes. During positive mode, the fragmentation of **1** yielded a product ion at *m/z* 315.1586 which in turn lost 18 Da followed by loss of 30 Da and ultimately neutral loss of 146 Da was seen. During negative mode, fragment ion at *m/z* 300.1371 was generated by the loss of 101 Da followed by continuous loss of methyl radical.

To further confirm the new structures, ESI-MS/MS experiment of compound **1** was carried out. The positive and negative modes of compound **1** were achieved by MS^2^ experiments (see Supplementary Material). The positive ion ESI mass spectrum of compound gave the major [M+H]^+^ ion at *m/z* 403.2101. As shown in **Figure 5**, the fragmentation of this precursor ion yielded an interesting product ion at *m/z* 315.1586, which was attributed to the elimination of a neutral molecule C_4_H_8_O_2_ (88 Da) from the precursor ion *m/z* 403.2101. The product ions at *m/z* 297.1478 and 285.1474 were generated by the loss of 18 and 30 Da, which were reasonably assigned as the elimination of H_2_O and formaldehyde (CH_2_O), respectively. Furthermore, neutral loss of 146 Da (assigned to C_11_H_14_) to produce fragment ion at *m/z* 139.0851 was also observed. The negative ion ESI mass spectrum of the compound gave the major [M-H]^-^ ion at *m/z* 401.1984. The fragment ion at *m/z* 300.1371 was generated by the loss of 101 Da, which was consistent with the presence of the hydroxyl residue at C-3 position with continuous loss of methyl radical (15 Da) to yield a product ion at *m/z* 285.1122.

**FIGURE 6:**
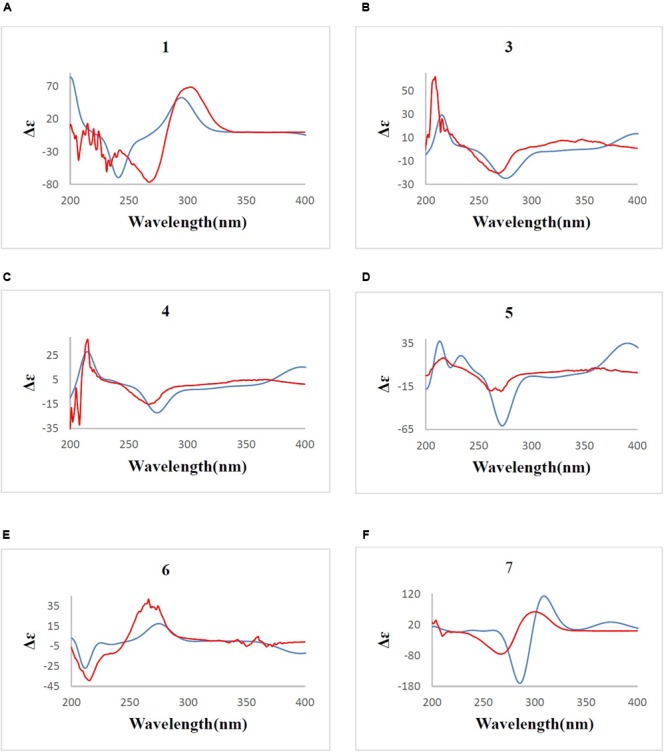
**(A–F)** B3LYP/6-31+G(d,p) calculated ECD spectrum (blue) and the experimental ECD spectrum (red) (σ = 0.2 eV) for compounds **1, 3, 4, 5, 6, 7**. The comparison of the calculated and experimental ECD spectra allowed the determination of the absolute configurations of compounds **1, 3, 4, 5, 6, 7**.

Determination of the absolute configuration of **1** by simulation of the ECD spectrum was performed. GAUSSIAN 09 was run to optimize the minimum energy geometries of two conformers using DFT at the B3LYP/6-31+G(d,p) level in the gas phase. The ECD spectra calculation were simulated using the time-dependent density functional theory (TDDFT) employing the B3LYP functional at the B3LYP/6-31+G(d,p) level in methanol (Frisch et al., 2009; Xie et al., 2011, 2012; Bruhn et al., 2015). SpecDis was used to draw the calculated ECD curves with a σ of 0.2 eV. The calculated ECD spectrum was seen to be identical to the experimental ECD spectrum where the absolute configuration of **1** was determined as 2*S*, 3*S*, 4*S*, 5*S* (**Figure 6**). Compound **1**, being a new polyketide was thus termed as neocitreoviridin (**Figure 2**).

#### Compound 7

Polyene α-pyrone polyketide mycotoxins, a group of compounds attracted lots of attention because of their novel structures as well as biological properties (Wang et al., 2005) whose structure possess a conjugate pyrone system and a 2,6-dioxabicyclo[3.2.1]octane ring moieties. Two main structure features can be categorized as citreoviridinol analogs and aurovertin analogs (Nishiyama et al., 1987; Wang et al., 2005). The only difference between the two classes lies in that the 2,6-dioxabicyclo[3,2,1]octane ring is attached by ethyl group in the first category namely citreoviridinol analogs while methyl group is attached in aurovertin analogs. Aurovertins have been extensively studied whereas although citreoviridinol (four naturally occurring compounds have been isolated so far), were isolated during the year 1970–1980, the ^1^H and ^13^C NMR have not been assigned and their absolute configurations have not been achieved. During this study, three known citreoviridinol analogs namely citreoviridinol (**6**), epicitreoviridinol (**3**), and epiisocitreoviridinol (**4**) (Nishiyama et al., 1987; Wang et al., 2005) and one new citreoviridinol analog namely 10*Z*-isocitreoviridinol (**5**) were isolated as stressed metabolites. Their NMR assignments and the absolute configurations were achieved for the first time.

**FIGURE 7:**
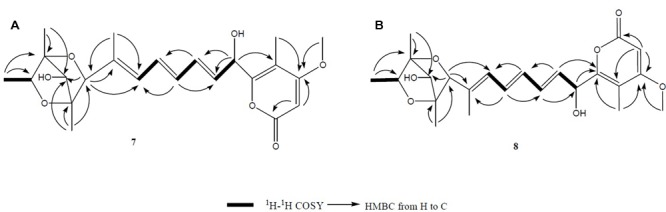
**(A,B)** The key ^1^H-^1^H COSY and HMBC correlations of compounds **7** and **8**. The olefinic chain was linked to α-pyrone ring by an oxygenated carbon which was confirmed by the ^1^H-^1^H COSY and HMBC. Moreover, a 2,5-dioxabicyclo[2.2.1]heptane ring was seen to be connected to a migrated polyene chain by detailed analysis of 2D NMR data of **7** and **8**.

**FIGURE 8:**
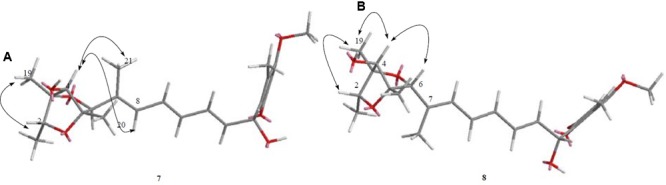
**(A,B)** The key NOESY correlations of compounds **7** and **8**. **(A)** The olefinic chain was assigned as β-oriented due to the observation of the NOESY cross peaks of H-4/Me-21 and H-4/H-8 allowing the determination of the relative configuration of **7**. **(B)** The diagnostic NOESY cross peak of H-4/H-6 as well as the NOESY cross peak of H-4/Me-19 demonstrated the configuration of **8**, which was different from **7**.

Compound **7** (**Figure 2**) was obtained as yellowish syrup. The molecular formula of **7** was determined to be C_23_H_30_O_7_ on the basis of HR-TOF-MS ion peak at *m/z* 419.2045 [M+H]^+^ (calcd. 419.2064). The ^1^H and ^13^C NMR spectra of **7** (**Table 3**) showed similar chemical shifts for seven carbon atoms of α-pyrone moiety as compared to the new compound **1** and known compounds citreoviridinol (**6**), epicitreoviridinol (**3**), and epiisocitreoviridinol (**4**) indicating that compound **7** is an α-pyrone polyketide. However, the polyene olefinic chain was proved to be different from those of citreoviridinol, aurovertin, and citreoviridin analogs (Wang et al., 2005; da Rocha et al., 2015) based on 1D and 2D NMR data of **7**. The unique proton signal at δ_H_ 5.24 (d, *J* = 6.85 Hz, H-13) in the ^1^H NMR spectrum of **7** differentiated its structure from all the naturally occurring citreoviridinol, aurovertin, and citreoviridin analogs where their olefinic chain is directly attached on an α-pyrone unit. The substitute of hydroxyl group on C-13 enabled the migration of the polyene chain. To determine the polyene migration, detailed 2D NMR experiments were carried out. The olefinic chain in the molecule **7** was linked to α-pyrone ring by a oxygenated carbon which was confirmed by the ^1^H-^1^H COSY sequence of C8–C9 = C10–C11 = C12–CO and long range correlations from proton signal at δ_H_ 5.24 (d, *J* = 6.85 Hz, H-13) to the olefinic carbon signal δ_C_ 131.54 (CH, C-12) in the polyene chain and the olefinic carbon signal δ_C_ 160.41 (C, C-14) in the α-pyrone ring. This is the first example of the polyene migration phenomenon in the α-pyrone polyketide. Because of the migration of the double bonds, the 2,6-dioxabicyclo[3,2,1]octane ring system was rearranged. The rearrangement of the ring system in the other side of the polyene chain of compound **7** was determined mainly by the analysis of HMBC and NOESY spectra. Since one double bond was migrated to C-7, the octane ring that was linked by oxygen to C-7 was opened on the linkage between oxygen atom and C-7. In spite of the octane ring opening, the NOESY correlations from the proton signal at δ_H_ 3.70 (s, H-4) to proton signal at δ_H_ 1.78 (s, Me-21) and olefinic proton signal at δ_H_ 6.18 (d, *J* = 11.39 Hz, H-8) indicated the closeness between them. Thus, it was hypothesized that the octane ring was condensed, which was confirmed by the analysis of the molecular formula C_23_H_30_O_7_. In the opened octane ring, there were two oxygenated carbons namely C-4 and C-6, holding the possibility to condense the ring with oxygenated C-3. However, the orientation of C_4_-O bond at C-4 was proved to point to the opposite side of C_3_-O bond owing to the NOESY cross peaks of the proton resonance at δ_H_ 3.70 (s, H-4) to the proton resonance at δ_H_ 6.18 (d, *J* = 11.39 Hz, H-8) and of the proton resonance at δ_H_ 3.70 (s, H-4) to the proton resonance at δ_H_ 1.78 (s, Me-21). This inference ruled out the possibility of rearranged ring between C-4 and C-3 by an oxygen atom bridge, leaving the only possibility to rearrange the 2,6-dioxabicyclo[3.2.1]octane ring into 2,5-dioxabicyclo[2.2.1]heptane ring bridged by O atom between C-6 and C-3. From the above established data, compound **7** was assigned as a pyrone polyketide with a 2,5-dioxabicyclo[2.2.1]heptane ring and a α-pyrone connected by a migrated polyene chain. The three main domains were constructed by the detailed analysis of 2D NMR data. The olefinic chain was deduced to connect the heptane ring through C6–C7 from the characteristic cross peaks Me-21/C-6, H-6/C-7, and H-6/C-8. The long range correlation from methyl proton signal at δ_H_ 1.78 (s, Me-21) to carbon signal at δ_C_ 128.70 (CH, C-8) indicated that Me-21 stayed at C-7 as those of citreoviridinols (Nishiyama et al., 1987; Forbes et al., 1991). Three methyl groups Me-1, Me-19, and Me-20 were positioned at C-2, C-3, and C-5, respectively on the 2,5-dioxabicyclo[2.2.1]heptane ring, which was inferred from the HMBC cross peaks of Me-1/C-2, Me-19/C-3, and Me-20/C-5. A hydroxyl group was placed on C-4 based on long range correlation from the proton signal at δ_H_ 3.70 (s, H-4) to carbon signal at δ_C_ 85.19 (C, C-3). The long range correlation from the proton resonance at δ_H_ 5.91 (dd, *J* = 15.10, 6.83 Hz, H-12) and the proton resonance at δ_H_ 5.24 (d, *J* = 6.85 Hz, H-13) in the polyene chain to the olefinic carbon signal at δ_C_ 160.41 (C, C-14) connected the polyene chain to the pyrone ring by an oxygenated C-13 on which a hydroxyl was placed. From the above analysis, the planar structure of **7** was elucidated as a new polyene pyrone polyketide as drawn in **Figure 7**. The relative configuration of **7** was determined by NOESY experiment. The olefinic chain was assigned as β-oriented due to the observation of the NOESY cross peaks of H-4/Me-21 and H-4/H-8. This also demonstrated that the hydroxyl group at C-4 was oriented as drawn in **Figure 8** which was confirmed by the observation of NOESY cross peaks H-4/Me-19 and H-4/Me-20. Me-1 was assigned as α-oriented as all known 2,6-dioxabicyclo[3.2.1]octane ring polyketides (Nishiyama et al., 1987; Forbes et al., 1991), based on NOESY correlations from the proton signal at δ_H_ 1.25 (H-2) to the proton signal at δ_H_ 1.27 (s, H-19). The polyketide **7** was thus given the trivial name penicillstressol.

Furthermore, the predicted ECD curve of **7** was calculated by quantum chemical methods at the [B3LYP/6-31+G(d)] level (Frisch et al., 2009; Xie et al., 2011, 2012; Bruhn et al., 2015) and the predicted ECD curve of **7** was similar to the experimental data. Thus the absolute configuration of **7** was determined as 2*R*, 3*S*, 4*R*, 5*S*, 6*S* (**Figure 6**).

#### Compound 8

Compound **8** (**Figure 2**), a yellowish syrup, was isolated as a mixture with compound **7**. The HRMS (TOF) exhibited an ion peak at *m/z* 419.2048 [M+H]^+^ (calcd. 419.2064), corresponding to the molecular formula C_23_H_30_O_7_. With the help of 2D NMR data of the mixture and the NMR data of the pure compound **7**, the proton signals and carbon signals were explicitly singled out (**Table 3**). The NMR data of compounds **8** and **7** were in very close agreement to each other with minor difference at C-5 (δ_C_ 85.90), C-6 (δ_C_ 89.28), C-20 (δ_C_ 15.42), C-21 (δ_C_ 13.69) and 5 ppm difference at C-3 (δ_C_ 80.27) in their ^13^C NMR spectra. Detailed 1D and 2D NMR experiments allowed the complete assignment of the planar structure of **8** which appeared to be the same as compound **7** (**Figure 7**). It was implied that the configuration of **8** in certain chiral centers was different between **8** and **7**. In order to clarify the difference between the structure features of the two compounds, detailed NOESY experiments were carried out. The hydroxyl group at C-4 was oriented toward the opposite side of the polyene chain as that in **7** due to the observation of NOESY cross peak H-4/Me-19 and H-4/H-6. The diagnostic cross peak of H-4/H-6 in the NOESY spectrum of **8** as opposed to the cross peak of H-4/Me-21 and H-4/H-8 in **7** demonstrated the configuration of **8** was different from **7** with the olefinic chain being β-oriented in **8** (**Figure 8**). The new compound was given the trivial name isopenicillstressol.

**FIGURE 9:**
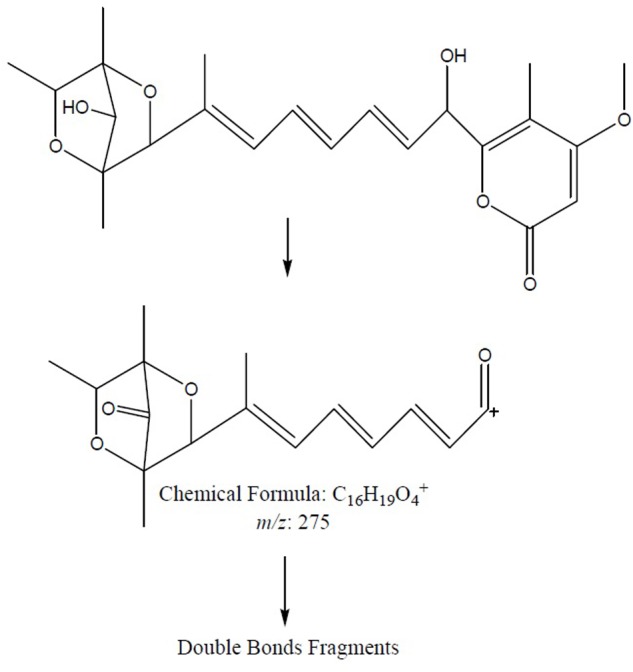
The plausible key MS^2^ fragmentation pathway of **7** and **8** was confirmed by ESI-MS/MS analysis. The characteristic ion at *m/z* 275.1261 was produced from precursor ion by the loss of C_7_H_8_O_3_ (140 Da) followed by double bonds fragmentation.

To further confirm the new structures, ESI-MS/MS experiment of compounds **7** and **8** was carried out. The positive mode of **7** and **8** was achieved by MS^2^ experiments with the precursor ion [M+H]^+^ ion at *m/z* 419. The characteristic ion at *m/z* 275.1261 was produced from precursor ion by the loss of a molecular of 4-methoxy-5-methyl-2H-pyran-2-one (140 Da, C_7_H_8_O_3_), which was the characteristic fragment ion in compounds **7** and **8** bearing a hydroxyl substitute in the aliphatic chain. The plausible MS^2^ fragmentation pathway was illustrated in **Figure 9**.

#### Compound 5

**FIGURE 10:**
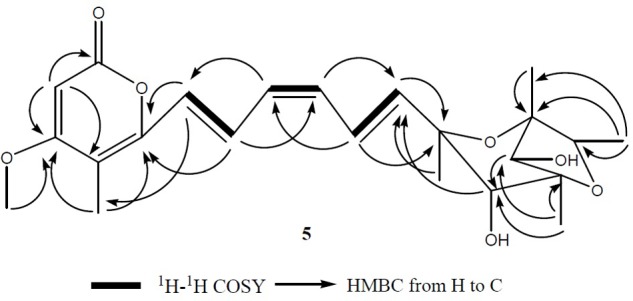
The key ^1^H-^1^H COSY and HMBC correlations of compound **5**. A 2,6-dioxabicyclo[3.2.1]octane attached to a α-pyrone through a conjugated olefinic chain was determined by analysis of ^1^H-^1^H COSY as well as long range correlation.

**FIGURE 11:**
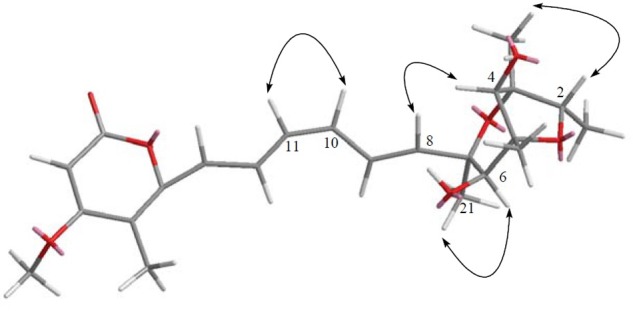
The key NOESY correlation of compound **5**. Detailed analysis of the conjugated olefinic chain with NOESY experiment demonstrated that the configuration of the double bond C10=C11 was *Z* configuration, thus was elucidated as 10*Z*-isocitreoviridinol.

Compound **5** was obtained as a yellowish syrup. The molecular formula C_23_H_30_O_7_ was determined by analysis of the HR-TOF-MS ion peak at *m/z* 419.2049 [M+H]^+^ (calcd. 419.2064). The formula was supported by ^13^C NMR data which indicated 9 degrees of unsaturation. In the ^1^H and ^13^C NMR spectrum of **5**, important characteristics signals such as methyl signals [δ_H_ 1.16 (d, *J* = 6.43 Hz, Me-1); δ_H_ 1.27 (s, Me-19); δ_H_ 1.29 (s, Me-20); δ_H_ 1.26 (s, Me-21); δ_H_ 2.00 (s, Me-22); δ_H_ 3.90 (s, Me-23) and δ_C_ 13.49 (CH3, C-1); δ_C_ 17.52 (CH3, C-19); δ_C_ 18.65 (CH3, C-20); δ_C_ 26.72 (CH3, C-21); δ_C_ 8.87 (CH3, C-22); δ_C_ 57.28 (CH3, C-23)] as well as conjugate olefinic pattern in the ^1^H-^1^H COSY (H-8/H-9, H-9/H-10, H-10/H-11, H-11/H-12, H-12/H-13) are indicative of a citreoviridinol backbone possessing a 2,6-dioxabicyclo[3.2.1]octane attached to a α-pyrone through a conjugated olefinic chain. Further detailed analysis of 2D NMR data and comparison of the NMR data with known citreoviridinol analogs in literature (Forbes et al., 1991; Wang et al., 2005) strongly suggested that the structure of compound **5** was highly similar to that of isocitreoviridinol (**Figure 10**). The NOESY cross peak from the proton signal at δ_H_ 3.98 (s, H-4) in the octane ring unit to the olefinic proton at δ_H_ 6.10 (H-8) revealed that the olefinic chain was β-oriented and a boat conformation of the octane was adopted because of the close distance between C-8 and C-4. The hydroxyl group at C-6 was deducted to be β-oriented from the analysis of a diagnostic cross peak from the proton signal at δ_H_ 3.60 (s, H-6) to the proton signal at δ_H_ 1.26 (s, Me-21). Me-19 displayed NOESY correlation with H-2 indicating an α-oriented Me-1. All above analysis matched well to the structure of the known compound isocitreoviridinol (Nishiyama et al., 1985). However, detailed analysis of the conjugated olefinic chain with NOESY experiment demonstrated that the configuration of the double bond C10=C11 was *Z* configuration as opposed to *E* configuration in isocitreoviridinol (**Figure 11**). Therefore, **5** was elucidated as a new compound named as 10*Z*-isocitreoviridinol.

In order to determine the stereochemistry of compound **5**, the theoretically calculated ECD spectrum and the experimental ECD spectrum were compared. The minimum energy geometries of two conformers were optimized using DFT at the B3LYP/6-31+G(d,p) level in the gaseous phase (Frisch et al., 2009; Xie et al., 2011, 2012; Bruhn et al., 2015). The calculated ECD spectrum was seen to be similar with the experimental ECD spectrum (**Figure 6**) due to the negative and positive Cotton effects (CEs) leading to the thorough configuration of **5**. Therefore the absolute configuration of **5** was determined as 2*S*, 3*R*, 4*S*, 5*R*, 6*S*, 7*R* (**Figure 2**).

### Known Compounds Elucidation

**FIGURE 12:**
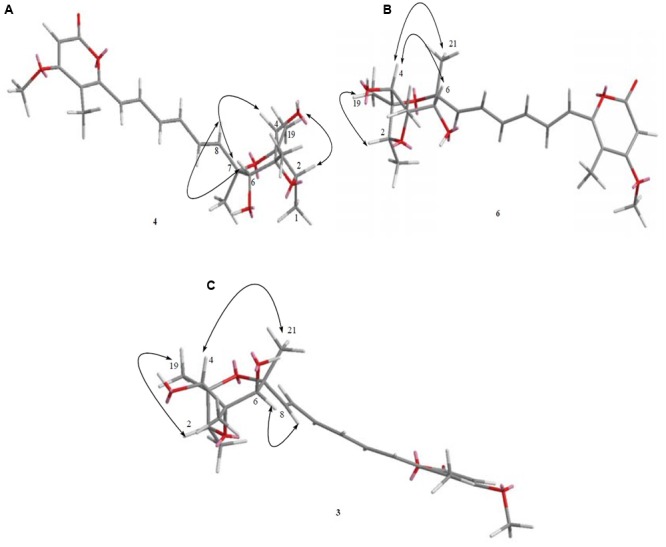
**(A–C)** The key NOESY correlation of known compounds **4, 6**, and **3** was determined where they were seen to possess the same planar structure.

Furthermore, known compounds epiisocitreoviridinol (**4**), citreoviridinol (**6**), epicitreoviridinol (**3**), and citreoviridin (**2**) (**Figure 2**) were determined by detailed 1D and 2D NMR data analysis as well as comparison of the data in literature (**Tables 1, 2, 4**). Since, known compounds epiisocitreoviridinol (**4**), citreoviridinol (**6**), epicitreoviridinol (**3**) possessed the same planar structure, NOESY experiments were applied to elucidate their structures as shown in **Figure 12**.

**Table 4:** ^1^H NMR data (500 MHz, δ in ppm, *J* in Hz), ^13^C NMR data (125 MHz, δ in ppm) for compounds **3** and **4** in CDCl_3_.

Position	3 (CDCl_3_)		4 (CDCl_3_)	
	δ_C_	δ_H_ (*J* in Hz)	δ_C_	δ_H_ (*J* in Hz)
1	13.09, CH_3_	1.20d (6.38)	13.18, CH_3_	1.19d (6.34)
2	78.07, CH	4.02^∗^	79.54, CH	4.08^∗^
3	84.01, C		82.97, C	
4	74.61, CH	3.98s	75.33, CH	3.98s
5	83.09, C		82.62, C	
6	82.77, CH	3.75s	80.29, CH	3.61s
7	76.42, C		78.18, C	
8	141.93, CH	6.22^∗^	146.64, CH	5.99d (15.33)
9	128.81, CH	6.39^∗^	128.14, CH	6.38^∗^
10	137.80, CH	6.53^∗^	137.25, CH	6.49^∗^
11	131.27, CH	6.48^∗^	131.53, CH	6.43^∗^
12	135.99, CH	7.16dd (14.99, 11.21)	135.84, CH	7.16dd (15.07, 11.09)
13	119.02, CH	6.51^∗^	119.22, CH	6.46^∗^
14	154.56, C		154.52, C	
15	108.12, C		108.36, C	
16	170.99, C		171.10, C	
17	88.66, CH	5.55s	88.72, CH	5.60s
18	164.43, C		164.63, C	
19	16.15, CH_3_	1.25s	17.28, CH_3_	1.25s
20	16.92, CH_3_	1.33s	18.57, CH_3_	1.34s
21	31.57, CH_3_	1.33s	26.50, CH_3_	1.28s
22	8.85, CH_3_	1.97s	8.85, CH_3_	1.98s
23	56.27, CH_3_	3.85s	56.31, CH_3_	3.86s

*^∗^Peaks are overlapped.*

The relative configuration of the known compound citreoviridin was also confirmed by NOESY experiments as shown in **Figure 4**.

The absolute configurations of the known compounds epiisocitreoviridinol (**4**) and citreoviridinol (**6**), epicitreoviridinol (**3**) were determined by simulation of the ECD spectra. GAUSSIAN 09 program was employed to optimize the minimum geometries of two conformers DFT at the B3LYP/6-31+G(d) level in the gas phase The ECD spectra calculation were simulated using the time-dependent density functional theory (TDDFT) using the B3LYP functional at the B3LYP/6-31+G(d) level in methanol. The calculated ECD curves were drawn by using SpecDis with a σ of 0.2 eV (Frisch et al., 2009; Xie et al., 2011, 2012; Bruhn et al., 2015). As such, the absolute configurations of epiisocitreoviridinol (**4**), citreoviridinol (**6**), and epicitreoviridinol (**3**) were determined as *2S, 3R, 4S, 5R, 6R, 7R* and *2R, 3S, 4R, 5S, 6S, 7R* and *2R, 3S, 4R, 5S, 6R, 7R*, respectively (**Figure 6**).

### Biological Activity, Cytotoxicity, and Possible Mode of Action

The isolated novel and known compounds were tested for their bacteriostatic as well as bactericidal abilities against three pathogenic bacteria namely MRSA, *P. aeruginosa*, and *K. pneumoniae*. The ESKAPE pathogens were used in this study due to fact that they are the main cause of nosocomial infections worldwide.

The novel compounds **7** and **8** exhibited impressive antibiotic abilities with MIC value of around 0.5 μg/mL followed by the novel compound **5** as well as the known compounds **3** and **6** which also showed potent antibiotic capacities with MIC values of 1 and 4 μg/mL against MRSA, respectively. Moreover, compounds **1, 7**, and **8** displayed strong antibiotic activities with MIC values of around 4 followed by MIC values of 8 for compounds **2** and **5** against *P. aeruginosa*. However, lower antibacterial activities were noted for compounds **1, 2**, and **4** against MRSA and compounds **3, 4**, and **6** against *P. aeruginosa* as well as compounds **1, 2, 5, 7**, and **8** against *K. pneumoniae* with MIC values of around 64 μg/mL. The positive control tetracycline displayed MIC values of around 2 and 8 μg/mL against MRSA and *P. aeruginosa*, respectively. The MBCs of the different potent compounds ranged from 1 to 128 μg/mL against MRSA, *P. aeruginosa*, and *K. pneumoniae*. The MIC and MBC values of the isolated compounds surpassing 128 μg/mL against the three pathogens were considered as “negligible capacity” (Supplementary Table S1).

Moreover, due to the lack of the amount of the different isolates, only compounds **7** and **8** were evaluated for their cytotoxicity. As a result, the new antibiotics **7** and **8** were not obviously harmful toward normal liver cell lines LO2, showing IC_50_ values above 100 μg/mL.

Various factors such as the difficulty of entering pathogenic cells where Gram-negative bacteria being famous for their tenacious outer membrane and cytoplasmic membrane as well as their efflux pump or the inactivation or alteration of the target in both Gram-positive and Gram-negative bacteria (Singh and Barett, 2006) have made it challenging to find potent antimicrobials. For instance, one among the mechanisms of β-lactams was revealed as being able to interrupt the homeostatic biosynthesis of cell walls. They showed the capacity of restricting peptide bond formation which are usually catalyzed by transpeptidase enzymes, thus hindering the cross-bonding of peptidoglycan units in pathogenic cell walls (Tipper and Strominger, 1965; Wise and Park, 1965) whereas quinolones/fluoroquinolone are known to constrain DNA gyrases or topoisomerase II and IV which are vital for strand rejoining (Chen et al., 1996; Drlica et al., 2008). On the other hand, macrolides displayed the ability of impeding protein synthesis by linking to the 50S bacterial ribosomal subunit leading to the restriction of the attachment of the peptidyl to the tRNA to the next amino acid by peptidyl transferase (Menninger and Otto, 1982; Vannuffel and Cocito, 1996; Kohanski et al., 2010).

The compounds isolated in this study belong to the α-pyrone class of antibiotics (Tupin et al., 2009) where the structures of the new antibiotics **7, 8**, and **5** bear similarities to myxopyronins which possess an α-pyrone and polyene units. In a previous study, myxopyronins were suggested as being RNA synthesis inhibitors. The main target of myxopyronins was the bacterial RNA polymerase (RNAP) which consists of large subunits of β and β′, giving RNAP a jaw-like shape. It was revealed that myxopyronin had the ability of binding to the switch-2 region of the RNAP β′ subunit, which restricted the open promoter complex formation. Moreover, it was also shown that myxopyronin had greater chance of hampering transcription at an early initiation step, most probably before the addition of the promoter DNA (Tupin et al., 2009). Thus, it could be hypothesized that the compounds exhibiting antibiotic capacities might have targeted the RNAP, which impeded interactions between RNAP and DNA suppressing the formation of an active open promoter complex during transcription as seen for myxopyronin in a study carried out by (Belogurov et al., 2008). Another study carried out by Mukhopadhyay et al. (2008), showed that myxopyronin had an impact on the opening and closing of the RNAP clamp, which avert the downstream DNA segment from entering the RNAP cleft. As a whole, it is plausible to postulate that the potent compounds isolated in this study could be prominent transcription inhibitors in pathogens. As a consequence, Gram-positive and Gram-negative bacteria have specific targets which need to be examined fastidiously and addressed by promising compounds exhibiting anti-pathogenic activities where the concept of “synergy between antibiotics” might also be an effective recourse for human health benefits.

## Conclusion

Antibiotics have long been on the first row in fighting against microbial infections. Due to evolution of pathogens leading to antibiotic resistance, researchers are encouraged to focus on the isolation of antibacterial compounds, their mechanisms of action as well as their biosynthetic pathway which have been neglected for quite some time. Microorganisms dwelling in harsh conditions like the hydrothermal vents bearing very high temperatures or the arctic regions with temperatures sometimes below 0°C, are exceptional places with unlimited promising secondary metabolites with antimicrobial capacities. Conventional techniques as applied before should be improved to yield potent metabolites with innovative pharmaceutical abilities. Henceforth, culturing the unculturable by applying different concept like optimization of laboratory conditions, coculture techniques or making use of various elicitors like ecological signals and chemical cues should be addressed to unveil the production of cryptic antibiotics (Zhu et al., 2014). For instance, microbes can be challenged and triggered to generate enthralling and priceless natural products. As demonstrated in this study, heavy metal strategy being used as an elicitor, here referring to the heavy metal cobalt which showed a distinct HPLC profile when compared to the normal one, is proven to have an impact on the secondary metabolism of the fungus strain *Penicillium* sp. BB1122, thus producing four novel compounds which revealed potent antibiotic properties against MRSA and *P. aeruginosa*. The bioactivity results obtained in this study revealed the remarkable ability of the novel compounds to expose their potential as antibacterials. Moreover, in-depth studies are required to scrutinize the action mechanism of the potent compounds against the ESKAPE pathogens which may bring to light some resistance secrets of clinical pathogens. As such, searching for effective elicitors may have a great impact on marine microbes which in turn lead to the generation of unexpected though potent natural products. This study may pave the way in researching new techniques for the isolation of novel secondary metabolites which can afterward be associated with cutting-edge molecular techniques to unlock cryptic biosynthetic gene clusters to streamline the discovery of novel pharmaceutically important compounds. As a whole, the ocean may be considered as a unique, untapped scaffold of marine drugs which can be considered as an exclusive shield against infectious diseases.

## Author Contributions

The experimental work was designed and performed by BA under the supervision of BW who is the corresponding author. XW and SH contributed analysis tools. BN, CP, NA, and BW wrote the paper.

## Conflict of Interest Statement

The authors declare that the research was conducted in the absence of any commercial or financial relationships that could be construed as a potential conflict of interest.
