# Supplementary material for: Stress-Driven Discovery of Novel Cryptic Antibiotics from a Marine Fungus Penicillium sp. BB1122
Source: Front Microbiol. 2017 Aug 3;8:1450. doi: 10.3389/fmicb.2017.01450 (PMC5540946; doi:10.3389/fmicb.2017.01450)
Supplement: Supplementary file 1 [file Data_Sheet_1.docx]

Supplementary Material

Stress-driven Discovery of Novel Cryptic Antibiotics from a Marine Fungus *Penicillium* sp. BB1122.

Bibi Nazia Auckloo^1^, Chengqian Pan^1^, Najeeb Akhter^1^, Bin Wu^1*^, Xiaodan Wu^2^, Shan He^3^

*** Bin Wu:** Corresponding Author: wubin@zju.edu.cn

# Supplementary Data

**Figure S1.** ^1^H NMR spectrum in CDCl_3_ for compound **1**.

**Figure S2.** ^13^C NMR spectrum in CDCl_3_ for compound **1**.

**Figure S3.** ^13^C NMR-2 spectrum in CDCl_3_ for compound **1**.

**Figure S4.** DEPT spectrum in CDCl_3_ for compound **1**.

**Figure S5.** COSY spectrum in CDCl_3_ for compound **1**.

**Figure S6.** COSY-2 spectrum in CDCl_3_ for compound **1**.

**Figure S7.** HMQC spectrum in CDCl_3_ for compound **1.**

**Figure S8.** HMQC-2 spectrum in CDCl_3_ for compound **1.**

**Figure S9.** HMBC spectrum in CDCl_3_ for compound **1**.

**Figure S10.** HMBC-2 spectrum in CDCl_3_ for compound **1**.

**Figure S11.** NOESY spectrum in CDCl_3_ for compound **1**.

**Figure S12.** ^1^H NMR spectrum in CDCl_3_ for compound **2**.

**Figure S13.** ^13^C NMR spectrum in CDCl_3_ for compound **2**.

**Figure S14.** DEPT spectrum in CDCl_3_ for compound **2.**

**Figure S15.** COSY spectrum in CDCl_3_ for compound **2**.

**Figure S16.** COSY-2 spectrum in CDCl_3_ for compound **2**.

**Figure S17.** HMQC spectrum in CDCl_3_ for compound **2**.

**Figure S18.** HMQC-2 spectrum in CDCl_3_ for compound **2**.

**Figure S19.** HMBC spectrum in CDCl_3_ for compound **2**.

**Figure S20.** HMBC-2 spectrum in CDCl_3_ for compound **2**.

**Figure S21.** HMBC-3 spectrum in CDCl_3_ for compound **2**.

**Figure S22.** HMBC-4 spectrum in CDCl_3_ for compound **2**.

**Figure S23.** NOESY spectrum in CDCl_3_ for compound **2**.

**Figure S24.** ^1^H NMR spectrum in CDCl_3_ for compound **3**.

**Figure S25.** ^13^C NMR spectrum in CDCl_3_ for compound **3**.

**Figure S26.** ^13^C NMR-2 spectrum in CDCl_3_ for compound **3**.

**Figure S27.** DEPT spectrum in CDCl_3_ for compound **3**.

**Figure S28.** NOESY spectrum in CDCl_3_ for compound **3**.

**Figure S29.** ^1^H NMR spectrum in CDCl_3_ for compound **4**.

**Figure S30.** ^13^C NMR spectrum in CDCl_3_ for compound **4**.

**Figure S31.** NOESY spectrum in CDCl_3_ for compound **4**.

**Figure S32.** ^1^H NMR spectrum in MeOD for compound **5**.

**Figure S33.** ^13^C NMR spectrum in MeOD for compound **5**.

**Figure S34.** ^13^C NMR-2 spectrum in MeOD for compound **5**.

**Figure S35.** DEPT spectrum in MeOD for compound **5**.

**Figure S36.** COSY spectrum in MeOD for compound **5**.

**Figure S37.** COSY-2 spectrum in MeOD for compound **5**

**Figure S38.** HMQC spectrum in MeOD for compound **5**.

**Figure S39.** HMQC-2 spectrum in MeOD for compound **5**.

**Figure S40.** HMQC-3 spectrum in MeOD for compound **5**.

**Figure S41.** HMBC spectrum in MeOD for compound **5**.

**Figure S42.** HMBC-2 spectrum in MeOD for compound **5**.

**Figure S43.** HMBC-3 spectrum in MeOD for compound **5**.

**Figure S44.** HMBC-4 spectrum in MeOD for compound **5**.

**Figure S45.** NOESY spectrum in MeOD for compound **5**.

**Figure S46.** ^1^H NMR spectrum in MeOD for compound **6**.

**Figure S47.** ^13^C NMR spectrum in MeOD for compound **6**.

**Figure S48.** DEPT spectrum in MeOD for compound **6**.

**Figure S49.** COSY spectrum in MeOD for compound **6**.

**Figure S50.** COSY-2 spectrum in MeOD for compound **6**.

**Figure S51.** HMQC spectrum in MeOD for compound **6**.

**Figure S52.** HMQC-2 spectrum in MeOD for compound **6**.

**Figure S53.** HMQC-3 spectrum in MeOD for compound **6**.

**Figure S54.** HMBC spectrum in MeOD for compound **6**.

**Figure S55.** HMBC-2 spectrum in MeOD for compound **6**.

**Figure S56.** HMBC-3 spectrum in MeOD for compound **6**.

**Figure S57.** HMBC-4 spectrum in MeOD for compound **6**.

**Figure S58.** NOESY spectrum in MeOD for compound **6**.

**Figure S59.** ^1^H NMR spectrum in MeOD for compound **7**.

**Figure S60.** ^13^C NMR spectrum in MeOD for compound **7**.

**Figure S61.** ^13^C NMR-2 spectrum in MeOD for compound **7**.

**Figure S62.** DEPT spectrum in MeOD for compound **7**.

**Figure S63.** COSY spectrum in MeOD for compound **7**.

**Figure S64.** COSY-2 spectrum in MeOD for compound **7**.

**Figure S65.** COSY-3 spectrum in MeOD for compound **7**.

**Figure S66.** HMQC spectrum in MeOD for compound **7**.

**Figure S67.** HMQC-2 spectrum in MeOD for compound **7**.

**Figure S68.** HMQC-3 spectrum in MeOD for compound **7**.

**Figure S69.** HMBC spectrum in MeOD for compound **7**.

**Figure S70.** HMBC-2 spectrum in MeOD for compound **7**.

**Figure S71.** HMBC-3 spectrum in MeOD for compound **7**.

**Figure S72.** NOESY spectrum in MeOD for compound **7**.

**Figure S73.** ^1^H NMR spectrum in MeOD for compound **8**.

**Figure S74.** ^13^C NMR spectrum in MeOD for compound **8**.

**Figure S75.** DEPT spectrum in MeOD for compound **8**.

**Figure S76.** COSY spectrum in MeOD for compound **8**.

**Figure S77.** COSY-2 spectrum in MeOD for compound **8**.

**Figure S78.** HMQC spectrum in MeOD for compound **8**.

**Figure S79.** HMQC-2 spectrum in MeOD for compound **8**.

**Figure S80.** HMQC-3 spectrum in MeOD for compound **8.**

**Figure S81.** HMBC spectrum in MeOD for compound **8**.

**Figure S82.** HMBC-2 spectrum in MeOD for compound **8**.

**Figure S83.** HMBC-3 spectrum in MeOD for compound **8**.

**Figure S84.** NOESY spectrum in MeOD for compound **8**.

**Figure S85.** HRTOFMS spectrum of compound **1**.

**Figure S86.** MS2 spectrum of compound **1** in positive mode.

**Figure S87.** MS2 spectrum of compound **1** in negative mode.

**Figure S88.** HRTOFMS spectrum of compound **2.**

**Figure S89**. HRTOFMS spectrum of compound **3**.

**Figure S90**. HRTOFMS spectrum of compound **4**.

**Figure S91**. HRTOFMS spectrum of compound **5**.

**Figure S92**. HRTOFMS spectrum of compound **6**.

**Figure S93.** HRTOFMS spectrum of compound **7**.

**Figure S94.** MS2 spectrum of compound **7** in positive mode.

**Figure S95.** HRTOFMS spectrum of compound **8**.

**Figure S96.** MS2 spectrum of compound **8** in positive mode.

**Figure S97.** IR spectra of compound **1**

**Figure S98.** IR spectra of compound **5**

**Figure S99.** IR spectra of compound **7**

**Figure S100.** IR spectra of compound **8**

**Figure S101.** B3LYP/6-31+G (d) optimized 3D structures & calculated ECD spectra of 1-1(red) and 1-2(green) of conformations of compound **1**.

**Figure S102.** B3LYP/6-31+G (d) optimized 3D structures & calculated ECD spectra of 3-1(red) and 3-2(green) of conformations of compound **3**.

**Figure S103.** B3LYP/6-31+G (d) optimized 3D structures & calculated ECD spectra of 4-1(red) and 4-2(green) of conformations of compound **4**.

**Figure S104.** B3LYP/6-31+G (d) optimized 3D structures & calculated ECD spectra of 5-1(red) and 5-2(green) of conformations of compound **5**.

**Figure S105.** B3LYP/6-31+G (d) optimized 3D structures & calculated ECD spectra of 6-1(red) and 6-2(green) of conformations of compound **6**.

**Figure S106.** B3LYP/6-31+G (d) optimized 3D structures & calculated ECD spectra of 7-1(red) and 7-2(black), 7-3(green) and 7-4(purple)of conformations of compound **7**.

**Figure S107.** Microscopic view of *Penicillium* sp. BB1122 (X400).

**Table S1.** Biological activities of compounds **1**-**8** (MIC and MBC values are given in µg/mL)

**Table S2.** 18S ribosomal RNA gene, partial sequence of *Penicillium* sp. BB1122

# Supplementary Figures and Tables

## Supplementary Figures

**Figure S1.** ^1^H NMR spectrum in CDCl_3_ for compound **1**.

**Figure S2.** ^13^C NMR spectrum in CDCl_3_ for compound **1**.

**Figure S3.** ^13^C NMR-2 spectrum in CDCl_3_ for compound **1**.

**Figure S4.** DEPT spectrum in CDCl_3_ for compound **1**.

**Figure S5.** COSY spectrum in CDCl_3_ for compound **1**.

**Figure S6.** COSY-2 spectrum in CDCl_3_ for compound **1**.

**Figure S7.** HMQC spectrum in CDCl_3_ for compound **1.**

**Figure S8.** HMQC-2 spectrum in CDCl_3_ for compound **1.**

**Figure S9.** HMBC spectrum in CDCl_3_ for compound **1**.

**Figure S10.** HMBC-2 spectrum in CDCl_3_ for compound **1**.

**Figure S11.** NOESY spectrum in CDCl_3_ for compound **1**.

**Figure S12.** ^1^H NMR spectrum in CDCl_3_ for compound **2**.

**Figure S13.** ^13^C NMR spectrum in CDCl_3_ for compound **2**.

**Figure S14.** DEPT spectrum in CDCl_3_ for compound **2**.

**Figure S15.** COSY spectrum in CDCl_3_ for compound **2**.

**Figure S16.** COSY-2 spectrum in CDCl_3_ for compound **2**.

**Figure S17.** HMQC spectrum in CDCl_3_ for compound **2**.

**Figure S18.** HMQC-2 spectrum in CDCl_3_ for compound **2**.

**Figure S19.** HMBC spectrum in CDCl_3_ for compound **2**.

**Figure S20.** HMBC-2 spectrum in CDCl_3_ for compound **2**.

**Figure S21.** HMBC-3 spectrum in CDCl_3_ for compound **2**.

**Figure S22.** HMBC-4 spectrum in CDCl_3_ for compound **2**.

**Figure S23.** NOESY spectrum in CDCl_3_ for compound **2**.

**Figure S24.** ^1^H NMR spectrum in CDCl_3_ for compound **3**.

**Figure S25.** ^13^C NMR spectrum in CDCl_3_ for compound **3**.

**Figure S26.** ^13^C NMR-2 spectrum in CDCl_3_ for compound **3**.

**Figure S27.** DEPT spectrum in CDCl_3_ for compound **3**.

**Figure S28.** NOESY spectrum in CDCl_3_ for compound **3**.

**Figure S29.** ^1^H NMR spectrum in CDCl_3_ for compound **4**.

**Figure S30.** ^13^C NMR spectrum in CDCl_3_ for compound **4**.

**Figure S31.** NOESY spectrum in CDCl_3_ for compound **4**.

**Figure S32.** ^1^H NMR spectrum in MeOD for compound **5**.

**Figure S33.** ^13^C NMR spectrum in MeOD for compound **5**.

**Figure S34.** ^13^C NMR-2 spectrum in MeOD for compound **5**.

**Figure S35.** DEPT spectrum in MeOD for compound **5**.

**Figure S36.** COSY spectrum in MeOD for compound **5**.

**Figure S37.** COSY-2 spectrum in MeOD for compound **5**.

**Figure S38.** HMQC spectrum in MeOD for compound **5**.

**Figure S39.** HMQC-2 spectrum in MeOD for compound **5**.

**Figure S40.** HMQC-3 spectrum in MeOD for compound **5**.

**Figure S41.** HMBC spectrum in MeOD for compound **5**.

**Figure S42.** HMBC-2 spectrum in MeOD for compound **5**.

**Figure S43.** HMBC-3 spectrum in MeOD for compound **5**.

**Figure S44.** HMBC-4 spectrum in MeOD for compound **5**.

**Figure S45.** NOESY spectrum in MeOD for compound **5**.

**Figure S46.** ^1^H NMR spectrum in MeOD for compound **6**.

**Figure S47.** ^13^C NMR spectrum in MeOD for compound **6**.

**Figure S48.** DEPT spectrum in MeOD for compound **6**.

**Figure S49.** COSY spectrum in MeOD for compound **6**.

**Figure S50.** COSY-2 spectrum in MeOD for compound **6**.

**Figure S51.** HMQC spectrum in MeOD for compound **6**.

**Figure S52.** HMQC-2 spectrum in MeOD for compound **6**.

**Figure S53.** HMQC-3 spectrum in MeOD for compound **6**.

**Figure S54.** HMBC spectrum in MeOD for compound **6**.

**Figure S55.** HMBC-2 spectrum in MeOD for compound **6**.

**Figure S56.** HMBC-3 spectrum in MeOD for compound **6**.

**Figure S57.** HMBC-4 spectrum in MeOD for compound **6**.

**Figure S58.** NOESY spectrum in MeOD for compound **6**.

**Figure S59.** ^1^H NMR spectrum in MeOD for compound **7**.

**Figure S60.** ^13^C NMR spectrum in MeOD for compound **7**.

**Figure S61.** ^13^C NMR-2 spectrum in MeOD for compound **7**.

**Figure S62.** DEPT spectrum in MeOD for compound **7**.

**Figure S63.** COSY spectrum in MeOD for compound **7**.

**Figure S64.** COSY-2 spectrum in MeOD for compound **7**.

**Figure S65.** COSY-3 spectrum in MeOD for compound **7**.

**Figure S66.** HMQC spectrum in MeOD for compound **7**.

**Figure S67.** HMQC-2 spectrum in MeOD for compound **7**.

**Figure S68.** HMQC-3 spectrum in MeOD for compound **7**.

**Figure S69.** HMBC spectrum in MeOD for compound **7**.

**Figure S70.** HMBC-2 spectrum in MeOD for compound **7**.

**Figure S71.** HMBC-3 spectrum in MeOD for compound **7**.

**Figure S72.** NOESY spectrum in MeOD for compound **7**.

**Figure S73.** ^1^H NMR spectrum in MeOD for compound **8**.

**Figure S74.** ^13^C NMR spectrum in MeOD for compound **8**.

**Figure S75.** DEPT spectrum in MeOD for compound **8**.

**Figure S76.** COSY spectrum in MeOD for compound **8**.

**Figure S77.** COSY-2 spectrum in MeOD for compound **8**.

**Figure S78.** HMQC spectrum in MeOD for compound **8**.

**Figure S79.** HMQC-2 spectrum in MeOD for compound **8**.

**Figure S80.** HMQC-3 spectrum in MeOD for compound **8**.

**Figure S81.** HMBC spectrum in MeOD for compound **8**.

**Figure S82.** HMBC-2 spectrum in MeOD for compound **8**.

**Figure S83.** HMBC-3 spectrum in MeOD for compound **8**.

**Figure S84.** NOESY spectrum in MeOD for compound **8**.


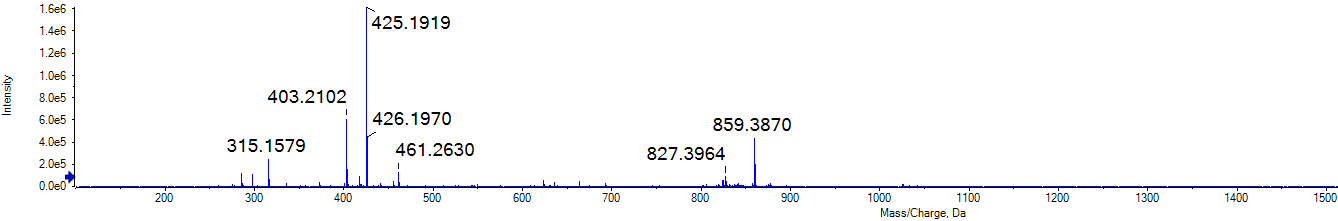
**Figure S85.** HRTOFMS spectrum of compound **1**.


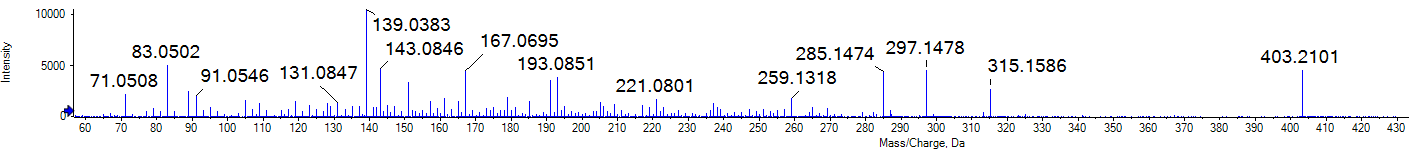


**Figure S86.** MS^2^ spectrum of compound **1** in positive mode.


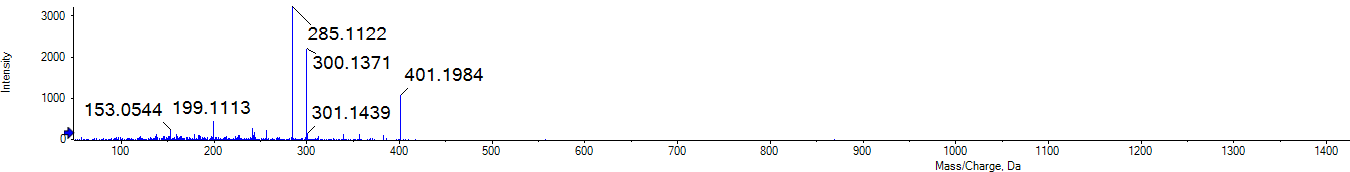


**Figure S87.** MS^2^ spectrum of compound **1** in negative mode.


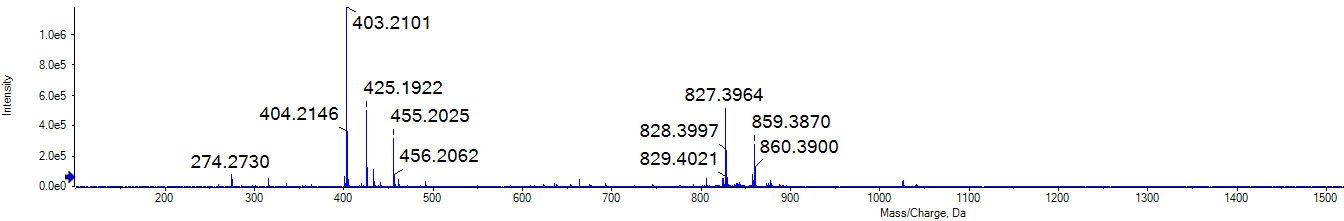


**Figure S88.** HRTOFMS spectrum of compound **2.**


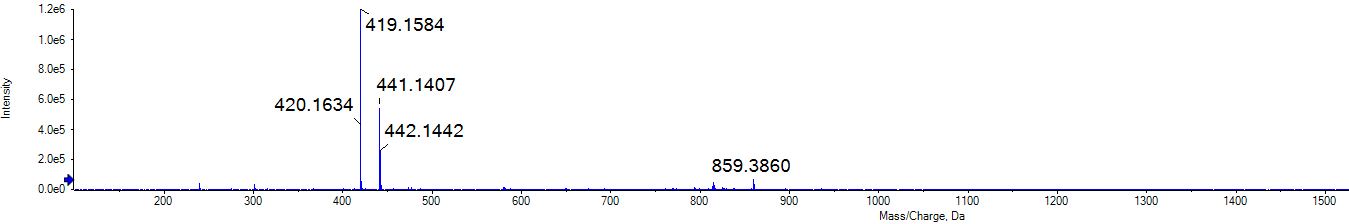


**Figure S89**. HRTOFMS spectrum of compound **3**.


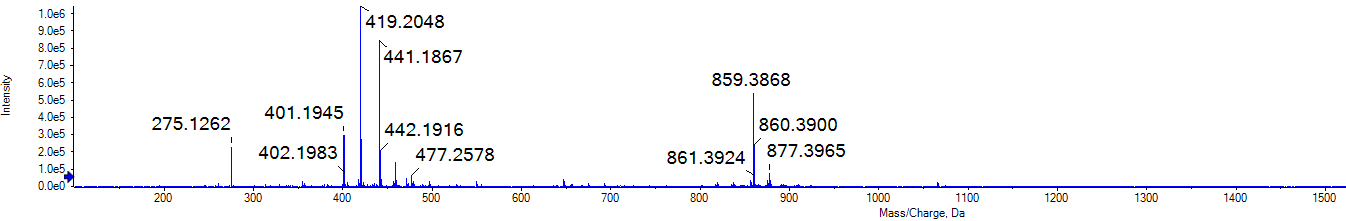


**Figure S90**. HRTOFMS spectrum of compound **4**.


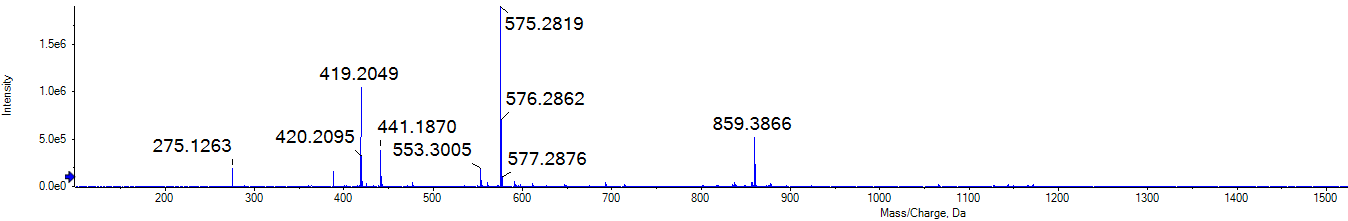


**Figure S91**. HRTOFMS spectrum of compound **5**.


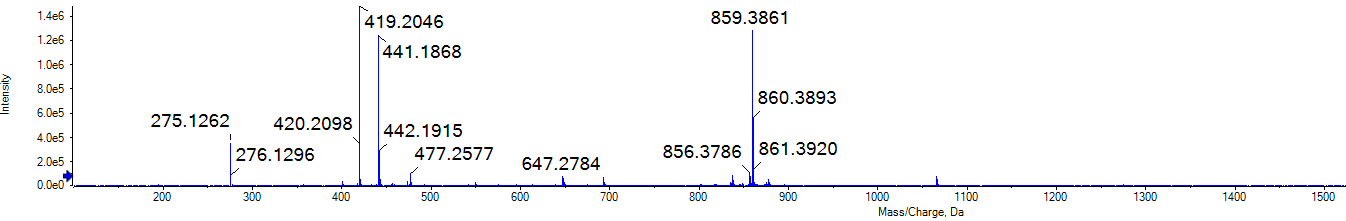


**Figure S92**. HRTOFMS spectrum of compound **6**.


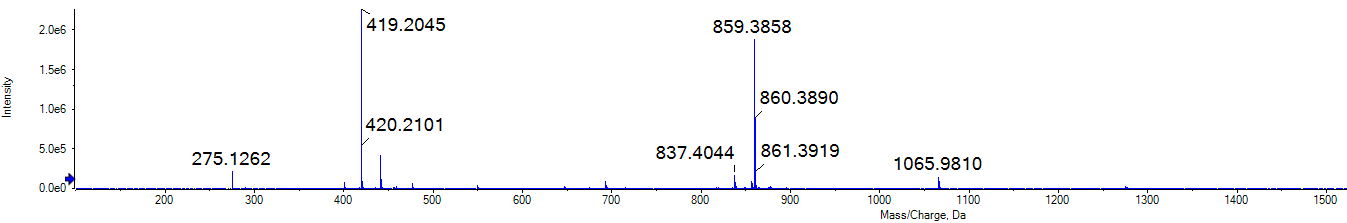


**Figure S93.** HRTOFMS spectrum of compound **7**.


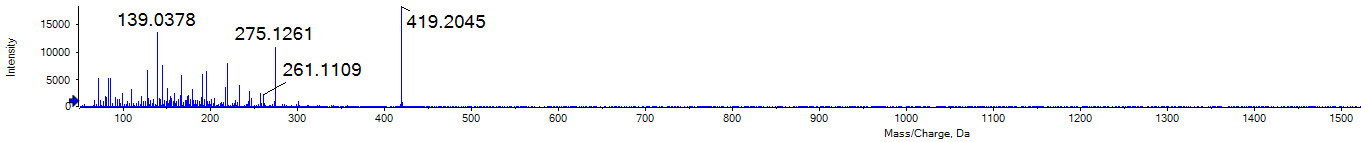


**Figure S94.** MS^2^ spectrum of compound **7** in positive mode.


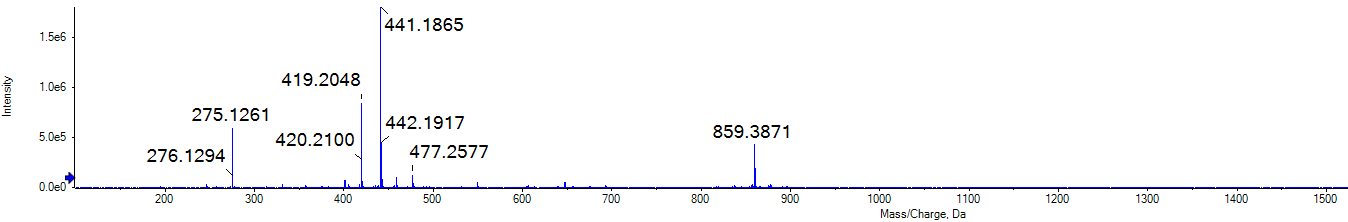


**Figure S95.** HRTOFMS spectrum of compound **8**.


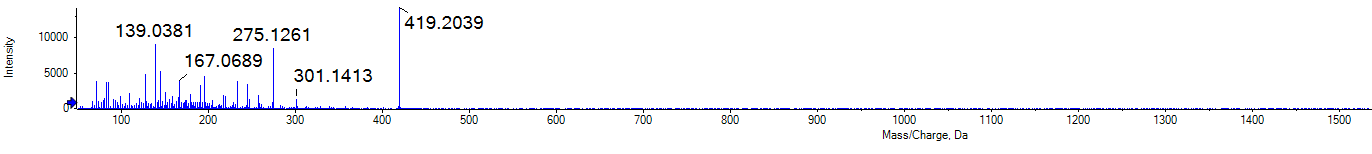


**Figure S96.** MS^2^ spectrum of compound **8** in positive mode.


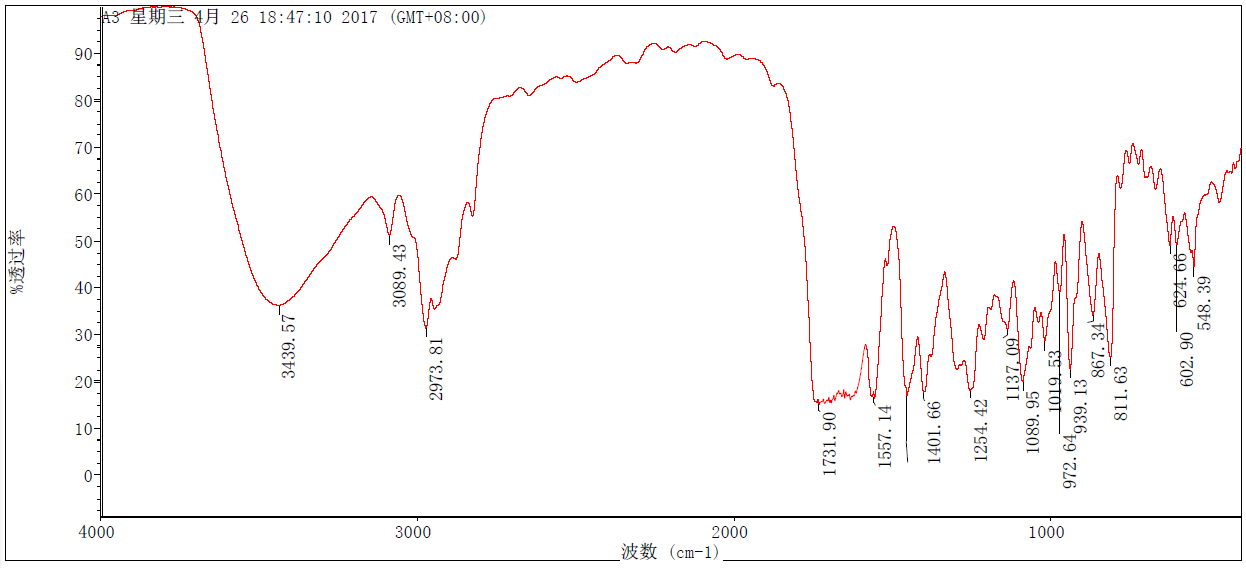


**Figure S97.** IR spectra of compound **1**


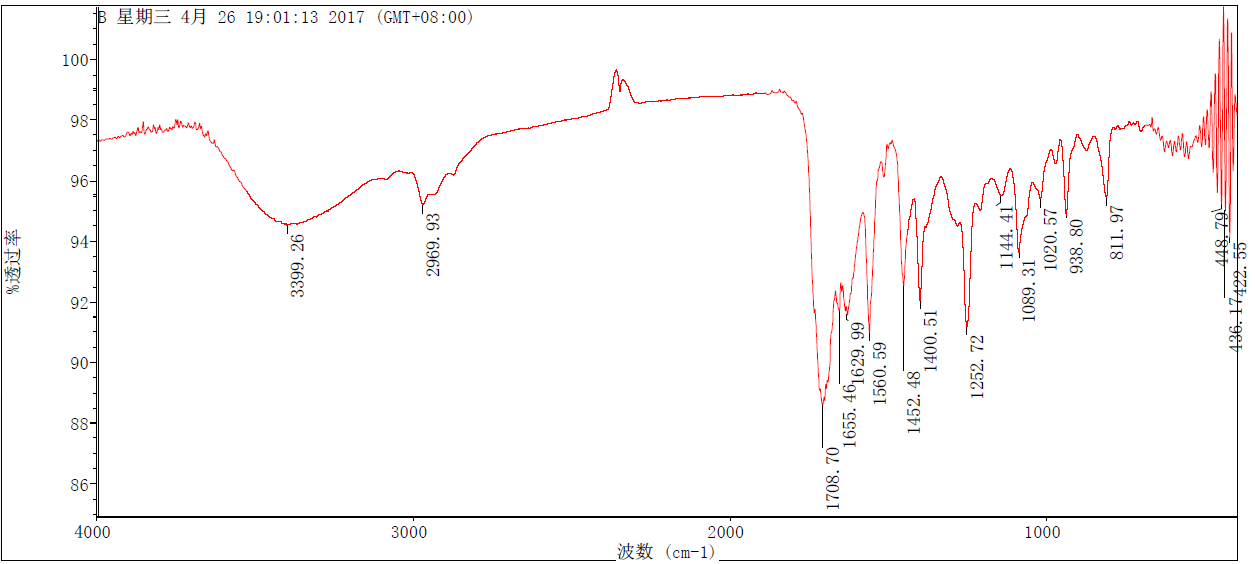


**Figure S98.** IR spectra of compound **5**


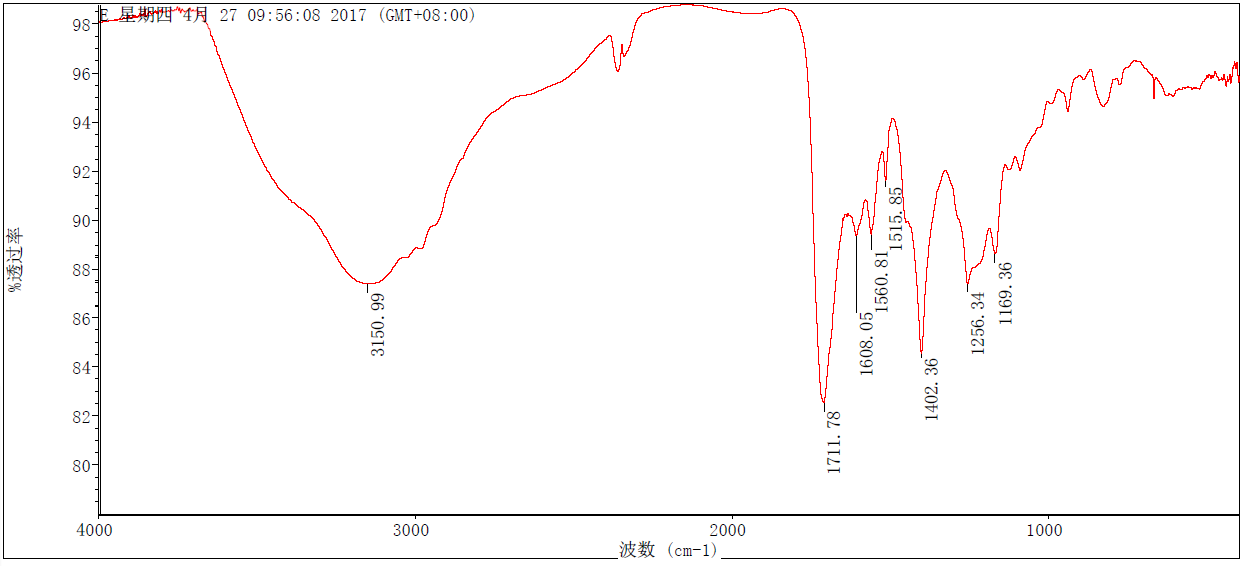


**Figure S99.** IR spectra of compound **7**


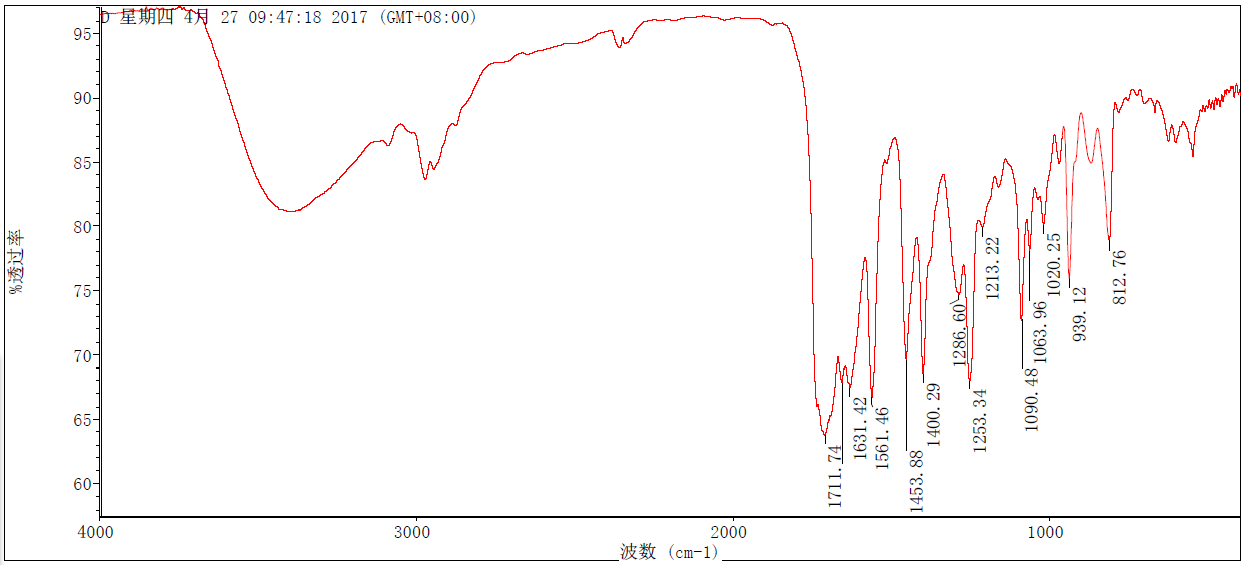


**Figure S100.** IR spectra of compound **8**

1-2

1-1


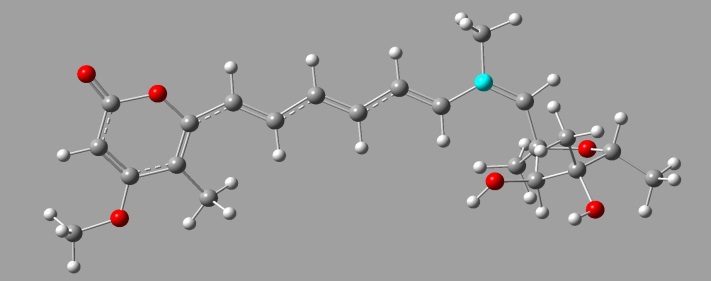

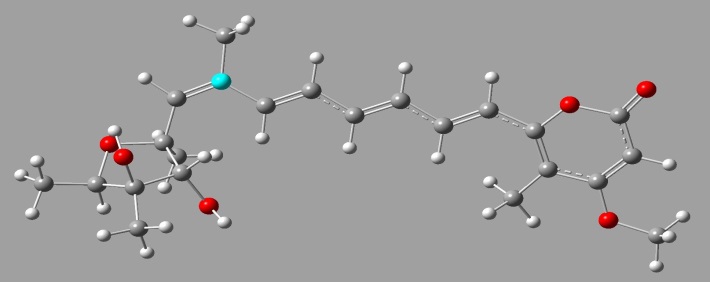


**Figure S101.** B3LYP/6-31+G (d) optimized 3D structures & calculated ECD spectra of 1-1(red) and 1-2(green) of conformations of compound **1**.

3-2

3-1


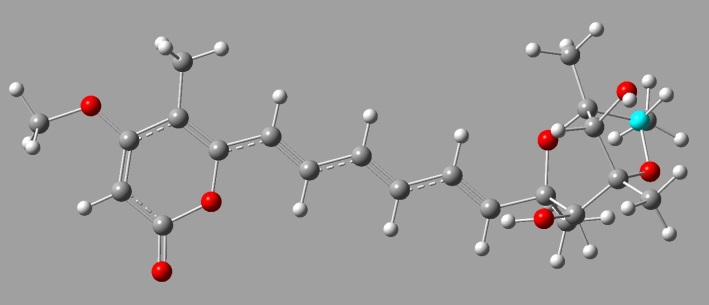

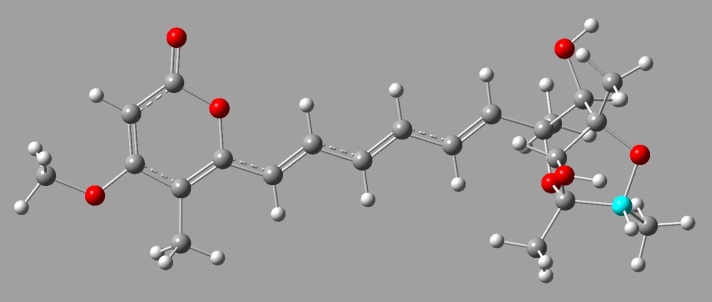


**Figure S102.** B3LYP/6-31+G (d) optimized 3D structures & calculated ECD spectra of 3-1(red) and 3-2(green) of conformations of compound **3**.

4-2

4-1


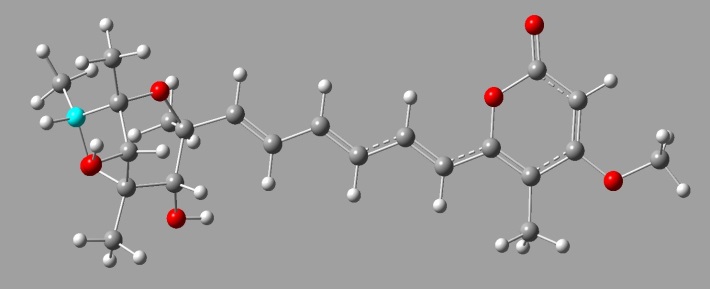

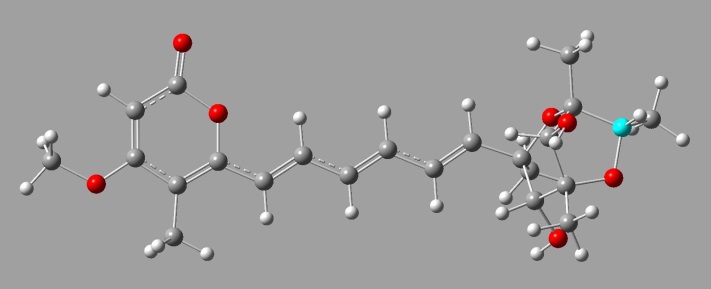


**Figure S103.** B3LYP/6-31+G (d) optimized 3D structures & calculated ECD spectra of 4-1(red) and 4-2(green) of conformations of compound **4**.

5-2

5-1


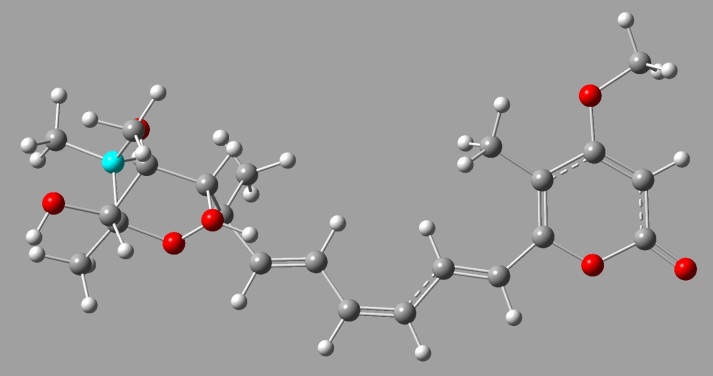

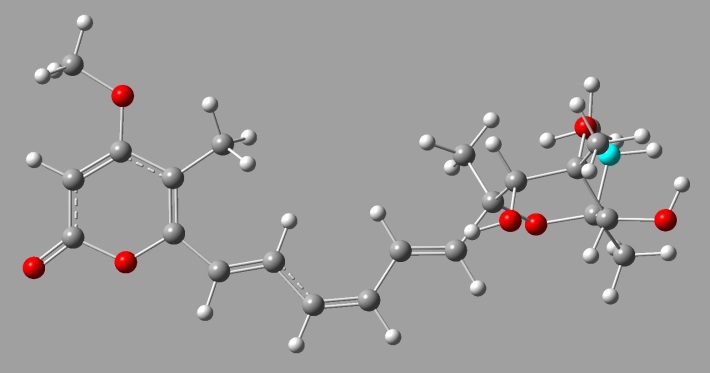


**Figure S104.** B3LYP/6-31+G (d) optimized 3D structures & calculated ECD spectra of 5-1(red) and 5-2(green) of conformations of compound **5**.

6-2

6-1


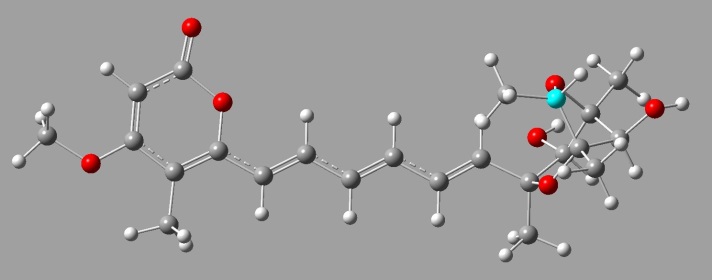

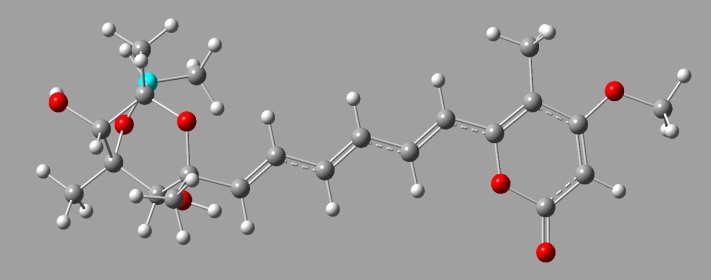


**Figure S105.** B3LYP/6-31+G (d) optimized 3D structures & calculated ECD spectra of 6-1(red) and 6-2(green) of conformations of compound **6**.

7-2

7-1


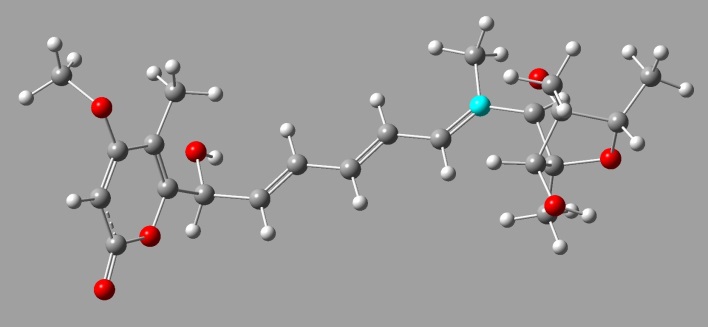

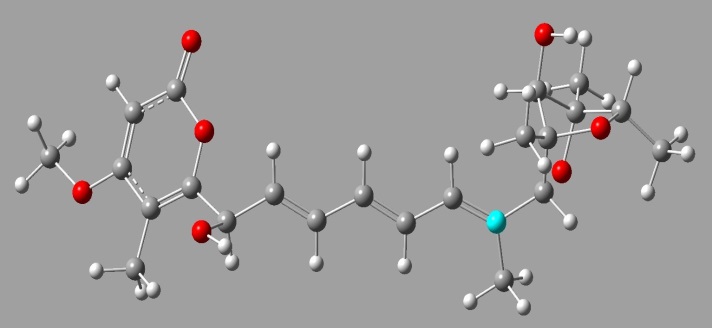


7-4

7-3


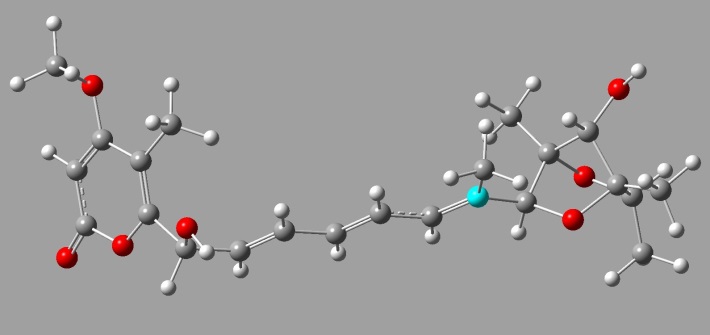

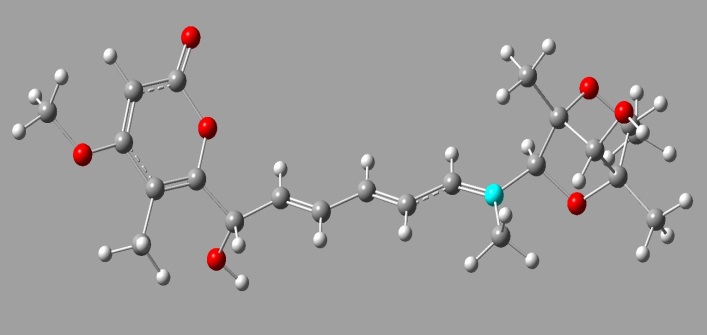


**Figure S106.** B3LYP/6-31+G (d) optimized 3D structures & calculated ECD spectra of 7-1(red) and 7-2(black), 7-3(green) and 7-4(purple)of conformations of compound **7**.

**
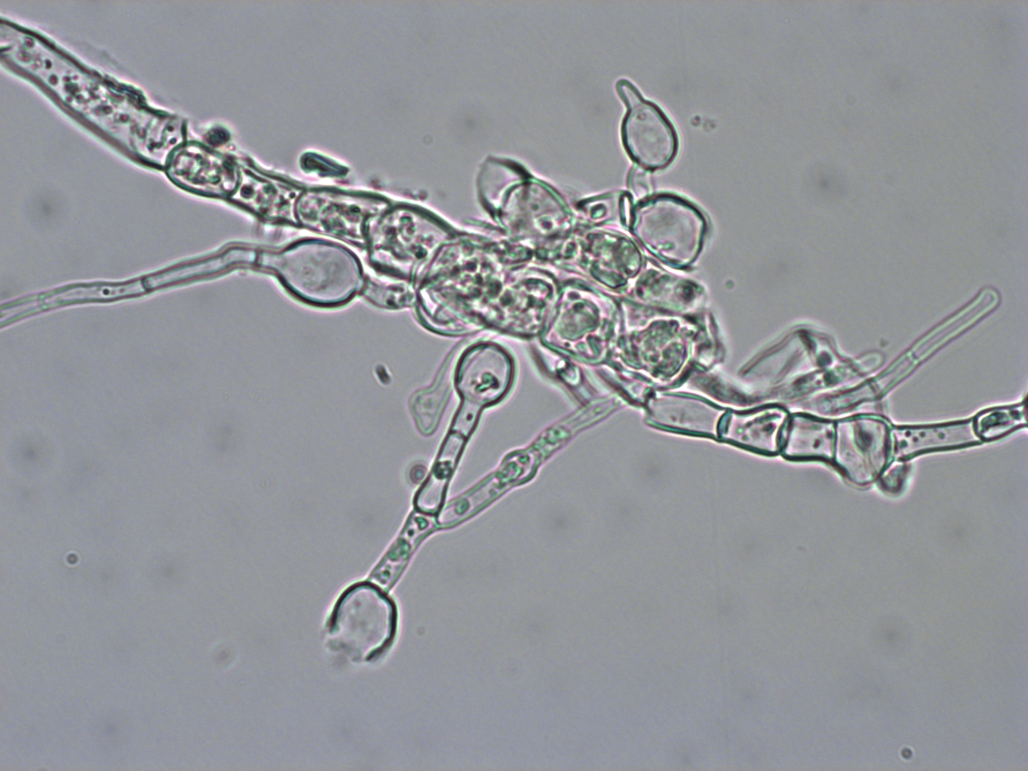
**

**Figure S107.** Microscopic view of *Penicillium* sp. BB1122 (X400µm)

## Supplementary Table

**Table S1.** Biological activities of compounds **1**-**8** (MIC and MBC values are given in µg/mL)

| **Compound** | **MRSA** | | ***Pseudomonas aeruginosa*** | | ***Klebsiella pneumoniae*** | |
| --- | --- | --- | --- | --- | --- | --- |
|  | **MIC** | **MBC** | **MIC** | **MBC** | **MIC** | **MBC** |
| **1** | 64 | 128 | 4 | 8 | 64 | 128 |
| **2** | 64 | 128 | 8 | 16 | 64 | 128 |
| **3** | 1 | 2 | 64 | 128 | - | - |
| **4** | 64 | 128 | 64 | 128 | - | - |
| **5** | 1 | 2 | 8 | 16 | 64 | 128 |
| **6** | 4 | 8 | 64 | 128 | - | - |
| **7** | 0.5 | 1 | 4 | 8 | 64 | 128 |
| **8** | 0.5 | 1 | 4 | 8 | 64 | 128 |
| **Tetracycline** | 2 | 4 | 8 | 16 | 64 | 128 |

(-): MIC and MBC values >128 µg/mL

**Table S2.** 18S ribosomal RNA gene, partial sequence of *Penicillium* sp. BB1122

| Strains | 18S ribosomal RNA gene (partial sequences) |
| --- | --- |
| *Penicillium* sp BB1122 | TCCGTAGGTGAACCTGCGGAAGGATCATTACCGAGTGAGGGCCCTCTGGGTCCAACCTCCCACCCGTGTTTATCGTACCTTGTTGCTTCGGCGGGCCCGCCTCACGGCCGCCGGGGGGCATCCGCCCCCGGGCCCGCGCCCGCCGAAGACACACAAACGAACTCTTGTCTGAAGATTGCAGTCTGAGTACTTGACTAAATCAGTTAAAACTTTCAACAACGGATCTCTTGGTTCCGGCATCGATGAAGAACGCAGCGAAATGCGATAAGTAATGTGAATTGCAGAATTCAGTGAATCATCGAGTCTTTGAACGCACATTGCGCCCCCTGGTATTCCGGGGGGCATGCCTGTCCGAGCGTCATTGCTGCCCTCAAGCACGGCTTGTGTGTTGGGCTCTCGCCCCCCGCTTCCGGGGGGCGGGCCCGAAAGGCAGCGGCGGCACCGCGTCCGGTCCTCGAGCGTATGGGGCTTCGTCACCCGCTCTGTAGGCCCGGCCGGCGCCCGCCGGCGAACACCATCAATCTTAACCAGGTTGACCTCGGATCAGGTAGGGATACCCGCTGAACTTAAGCATATCAATAAGCGGAGGAA |

**
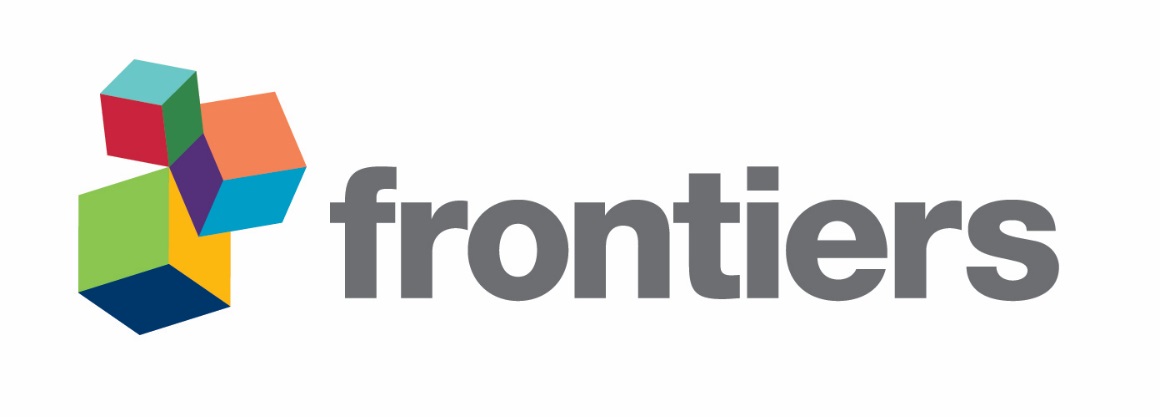
**
